# Mechanical forces trigger invasive behavior in synovial fibroblasts through N-cadherin/ADAM15 -dependent modulation of LncRNA H19

**DOI:** 10.1038/s41598-025-94012-2

**Published:** 2025-03-21

**Authors:** Tomasz Janczi, Beate Böhm, Yuliya Fehrl, Nikolas Hartl, Frank Behrens, Raimund W. Kinne, Harald Burkhardt, Florian Meier

**Affiliations:** 1 Division of Rheumatology, University Hospital Frankfurt, Goethe University Frankfurt am Main, 60590 Frankfurt am Main, Germany; 2 Fraunhofer Institute for Translational Medicine and Pharmacology ITMP, 60590 Frankfurt am Main, Germany; 3 Experimental Rheumatology Unit, Department of Orthopedics, Jena University Hospital, Waldkliniken Eisenberg GmbH, 07607 Eisenberg, Germany

**Keywords:** Cell biology, Rheumatology

## Abstract

Joint damage caused by immune-mediated inflammation in rheumatoid arthritis (RA) preferentially affects site-specific mechano-sensitive areas. The perception of physical forces in the synovial tissue by the residing fibroblasts initiates signalling responses with impact on cellular functions. Here, we describe a mechanotransduction pathway in rheumatoid arthritis synovial fibroblasts (RASF), which is critically dependent on the disintegrin metalloproteinase ADAM15 and N-cadherin (NCAD). Both molecules co-localize in NCAD-based adherens junctions and trigger mechanosignaling events involving the activation of p21-activated kinase 2 (PAK2). The mechanically induced phosphorylation of PAK2 subsequently leads to its co-recruitment together with the adaptor molecule Nck to the NCAD/ADAM15 complex at the cell membrane. These signal transduction events initiate strain-induced downregulation of lncRNA H19 and miR-130a-3p. They finally result in an upregulation of cadherin-11 (CDH11), thereby enhancing cell invasive properties - a feature characteristic of aggressive RASFs. Accordingly, we propose a new mechano-induced pathway that causes an altered composition of cadherin expression in the adherens junctions of synovial fibroblasts and likely contributes to the site-specific variability of the aggressive RASF-phenotype in RA-pathogenesis.

**Supplementary Information:**

The online version contains supplementary material available at 10.1038/s41598-025-94012-2.

## Introduction

Chronic inflammation in diarthrodial joints in rheumatoid arthritis (RA) is propagated by cells of the innate and adaptive immune system upon their immigration into the synovial membrane from the circulation, and by non-hematopoietic, tissue-resident synovial fibroblasts (RASFs). These RASFs, which are highly resistant to apoptosis, develop an RA- and site-specific aggressive phenotype characterized by invasive growth into cartilage and bone which is mediated by extracellular matrix-degrading enzymes^1^. Biomechanical loading at certain mechano-sensitive areas has been identified as an important factor controlling site-specific localization of inflammation and tissue damage, in part due to the load-induced upregulation of proinflammatory chemokines by synovial fibroblasts^2^.

The present study aims at elucidating signaling pathways induced under mechanical strain that may contribute to localization-specific erosivity of RASF in mechanosensitive compartments of the synovial membrane. In the course of these studies two cell surface proteins residing in adherens junctions N-cadherin (NCAD) and ADAM15, a disintegrin metalloproteinase strongly expressed in inflamed synovial membranes of RA patients^3^, proved to be crucial in a signaling pathway that involves the long noncoding RNA (lncRNA) H19. ADAM15 is a transmembrane protein with several extracellular domains, which binds intracellularly to SH2/SH3 domain-containing proteins, such as Src family kinases^4^ or the Nck adapter protein 1 (Nck1)^5^. ADAM15 functions as a mediator of anti-apoptotic signaling pathways triggered by various death stimuli, including FasL and genotoxic stress^6,7^.

LncRNAs, defined as RNAs > 200 nucleotides in length, are not translated into functional proteins and have key roles in gene regulation^8^. They can interact with nucleic acids and proteins regulating gene expression at multiple levels, including transcriptional, post-transcriptional and epigenetic regulation, and play pivotal roles in many (patho)-physiological processes, including tumorigenesis, osteogenesis, metabolism^9^ as well as inflammatory pathways in RA and osteoarthritis^10^. Here, our studies have revealed that mechanical stress induces NCAD- and ADAM15-dependent modulation of lncRNA H19. This lncRNA, with its known role as an oncogene that promotes cell growth, survival, migration, invasion and metastasis in a variety of malignant tumors^11^, was also described as being upregulated in RASFs in synovial tissue samples^12^. Mechanistically, H19 could function as a competing endogenous RNA (ceRNA) that acts as a molecular sponge for microRNAs (miRNA) via its miRNA binding sites, but is also able to regulate gene transcription via alternative mechanisms e.g. by binding to epigenetic regulators or transcription factors^11^.

MiRNAs, i.e. small, non-coding RNA molecules, are negative regulators of gene expression, which decrease the stability of target mRNAs through binding to complementary recognition elements on the 3’ UTR of mRNAs, thereby suppressing translation. Due to a proposed role of miRNAs in the pathogenesis of inflammatory rheumatic diseases their signatures have been investigated in the search for disease markers in the circulation. In a study of RA patients and their asymptomatic first-degree relatives miR-130a-3p could be identified as one of the significantly regulated miRNAs in differential expression profiles^13–15^. Interestingly, our in vitro studies uncovered a mechanosensitivity of miR-130a-3p in RASFs mediated by strain-induced downregulation of H19.

Moreover, a new gene target of miR-130a-3p could be identified: cadherin-11 (CDH11), a classical cadherin adhesion molecule that mediates homophilic cell-to-cell adhesion that is expressed by RASFs in addition to the mesenchymal NCAD^16^. CDH11 expression is essential for the organization of the synovial membrane consisting of a thin lining cell layer of condensed fibroblasts and co-compacted macrophages, which covers the loose connective tissue of the synovial sublining. Moreover, CDH11 expression in RASFs has been identified as being crucial for the RA-specific transformation of the synovial lining into an aggressive hyperplastic pannus tissue that attaches to, infiltrates and destroys articular cartilage and bone. Thus, cadherin-11 deficient mice develop a hypoplastic synovial lining, exhibit a disorganized synovial response to inflammatory challenge and are resistant to arthritis induction^17^.

In our studies, we unraveled a new mechanotransduction pathway orchestrated by NCAD/ADAM15 that converts mechanical strain into lncRNA H19/miR-130a-3p-mediated upregulation of CDH11 in RASFs, thereby promoting their viability and cell invasive properties. Accordingly, this mechano-induced mechanism leading to an altered cadherin expression pattern in the adherens junction of synovial lining cells may significantly contribute to the mechanosensitive site-specific variability of an aggressive phenotype expression of RASFs in RA-pathogenesis.

## Results

### Mechano-induced downregulation of LncRNA H19

**Fig. 1:**
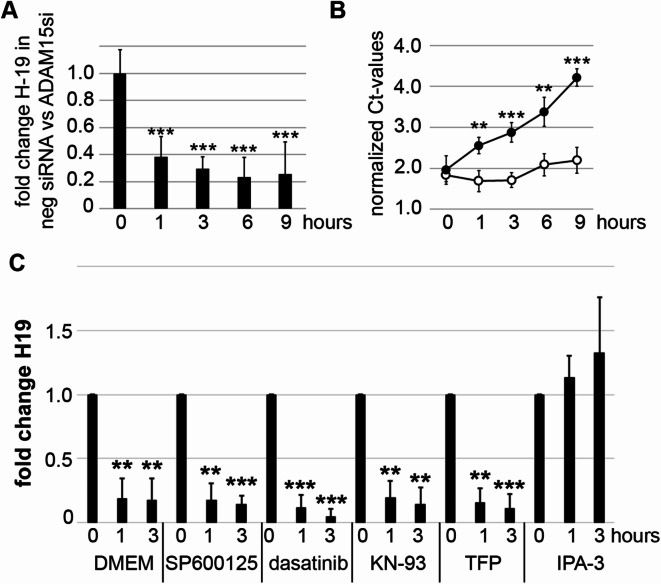
ADAM15-dependent mechano-induced downregulation of lncRNA H19 is affected by inhibition of p21-activated kinases (PAKs). (**A**,** B**) Synovial fibroblasts either silenced with an ADAM15 siRNA or a non-silencing negative siRNA were mechanically strained (1 Hz and 15% elongation) for 1–9 h and H19 levels determined by RT-qPCR. (**A**) Fold changes of H19 in ADAM15-expressing versus non-expressing cells were calculated using the 2^−ΔΔCt^. Shown is the mean ± SD of 6 different RASFs. (**B**) time course of GAPDH-normalized Ct values for H19 upon mechanical strain, showing increase of Ct values (i.e. lower H19 amounts) in ADAM15 expressing cells (black dots) compared to ADAM15 non-expressing cells (open circle) in one representative RASF cell line. (**C**) RT-qPCR for H19 of RASFs stimulated for 1 and 3 h in the presence of either DMEM medium, or inhibitors for JNK (SP600125), Src family kinases (dasatinib), CAMKII (KN-93), calmodulin (TFP) and PAKs (IPA-3). **p < 0.005, ***p < 0.0005, Student’s t-test, comparing ADAM15-expressing versus non-expressing cells or inhibitor versus DMEM.

Recently, we uncovered that mechanical strain resulted in the specific downregulation of 2 out of 372 disease-relevant lncRNAs, HOTAIR and H19, in rheumatoid arthritis synovial fibroblasts (RASFs)^18^. In order to confirm the mechano-induced and ADAM15-dependent downregulation of H19, RASF cell lines from 6 different donors were transfected with an ADAM15 siRNA or a negative control siRNA and H19 expression determined by RT-qPCR. A highly significant mechano-induced downregulation of H19 was detectable in ADAM15-expressing cells as compared to ADAM15-silenced cells, shown as fold change of H19 expression (Fig. 1A). Detailed analysis of H19 levels revealed that ADAM15-expressing RASFs downregulated H19 during mechanical strain throughout the whole loading period from 1 to 9 h. However, RASFs with silenced ADAM15 expression kept H19 at the same Ct levels throughout all time points, shown as the H19 Ct values for a representative donor cell line (Fig. 1B), clearly showing the ADAM15-dependent downregulation of H19 upon mechanical strain.

As the mechano-regulation of Hotair has previously been shown to be modulated by inhibition of JNK kinases^18^, we analyzed whether H19 regulation is affected by blocking diverse signaling pathways with inhibitors of JNK kinases (SP600125), Src and ABL family tyrosine kinases (dasatinib), Ca^2+^-signaling (CAMKII – KN93, calmodulin – TFP) and the p21-activated kinases (PAKs, IPA-3). RT-qPCR analysis showed that blocking major signaling pathways did not influence the mechano-induced downregulation of H19 (Fig. 1C). However, inhibition of PAKs with IPA-3 resulted in the reversal of this effect, indicating an involvement of PAK-signaling in mechano-induced H19 downregulation.

### ADAM15- and N-cadherin dependent mechano-induced activation of PAK2

**Fig. 2:**
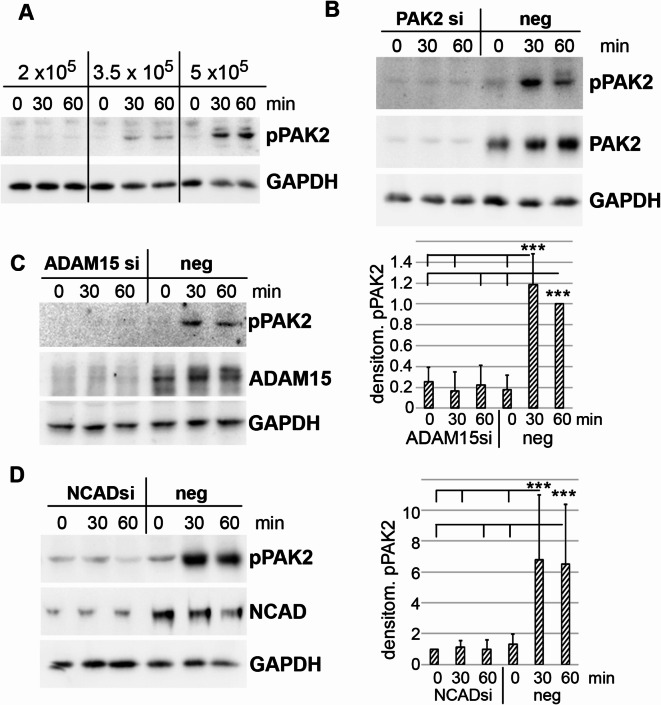
ADAM15- and N-cadherin (NCAD) dependent activation of PAK2 upon mechanical strain. (**A**–**D**) Immunoblots for pPAK2. (**A**) of RASFs seeded at different cell densities and mechanically strained for 30 and 60 min. (**B**) from strained RASFs with PAK2 silencing (si) and treated with a negative control siRNA (neg). (**C**,** D**) immunoblots for pPAK2, ADAM15 and NCAD from RASFs treated with ADAM15 and NCAD siRNA; right panels, showing the mean ± SD of densitometric analysis of 6 different RASF cell lines. ***p < 0.0005, Student’s t-test. GAPDH served as a loading control.

Next, we analyzed by immunoblotting, whether PAKs are activated by mechanical strain in RASFs. We discovered that PAK2 is phosphorylated at S20 in mechanically strained RASFs, which showed increased PAK2 activation with cells seeded at medium (3.5 × 10^5^/6-well) and high (5 × 10^5^/6-well) cell densities. However, PAK2 is not phosphorylated in cells seeded at low densities (2 × 10^5^/6-well) (Fig. 2A). Silencing of PAK2 with siRNA also confirmed the specific phosphorylation of PAK2 (Fig. 2B). Since cell density-dependent PAK2 activation implies triggering of adherens junctions, we investigated the role of NCAD, which is expressed in synovial fibroblasts^16^, and of ADAM15, which has been detected in endothelial adherens junctions^19^, in PAK activation. Expression of both ADAM15 and NCAD resulted in an induced and/or significantly increased mechano-activation of PAK2, whereas silencing of either ADAM15 or NCAD significantly reduced PAK2 phosphorylation (Fig. 2C, D), demonstrating that PAK2 activation is critically dependent on the presence of both ADAM15 and NCAD.

### Interaction of ADAM15 and N-cadherin (NCAD) is dependent on cell density

**Fig. 3:**
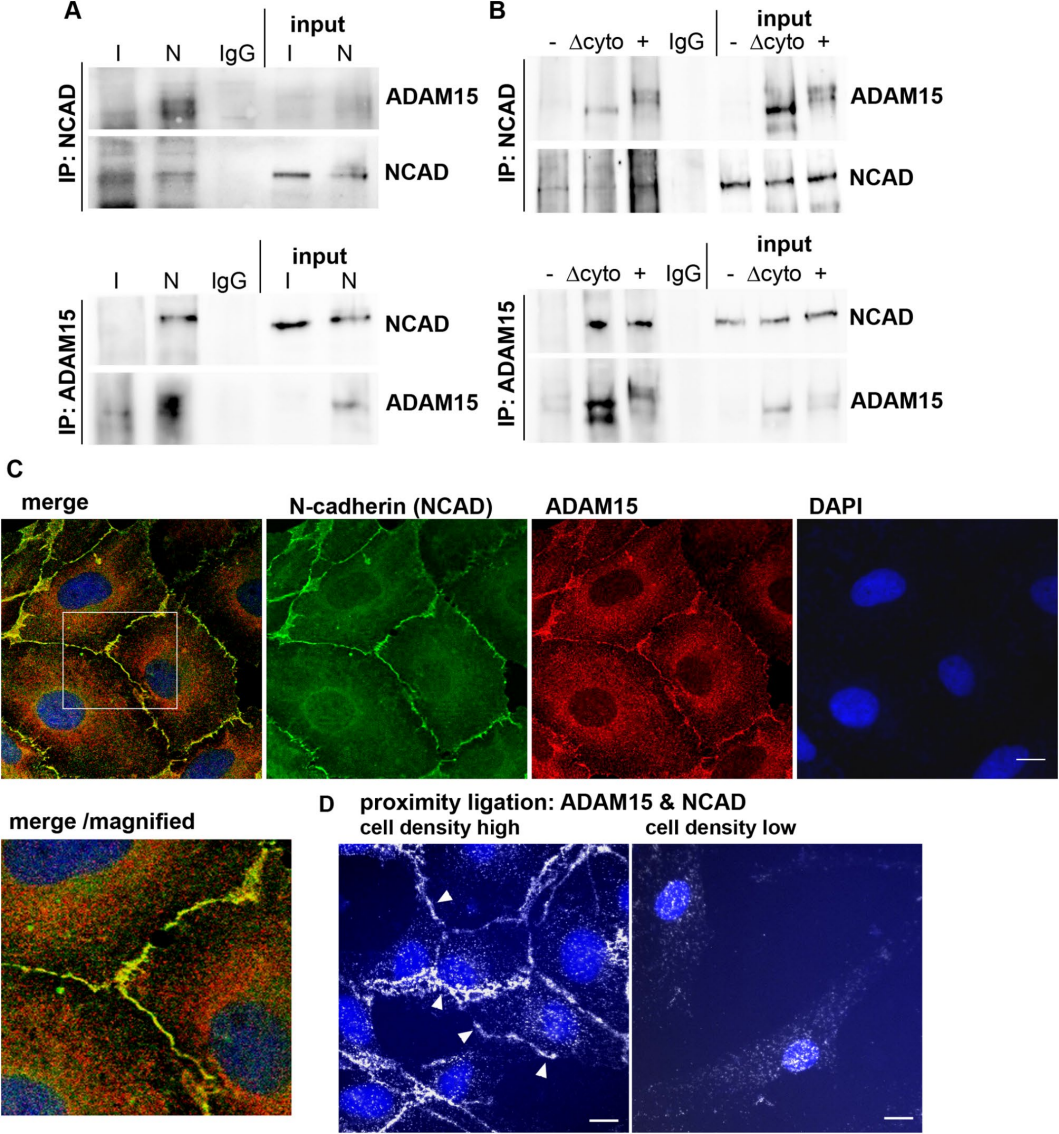
Interaction of ADAM15 with N-cadherin (NCAD) in synovial fibroblasts. (**A**) Co-immunoprecipitations (IP) of RASFs with prior silencing of ADAM15 using siRNA (I) and negative control siRNA (N) using NCAD antibodies (upper panels) or ADAM15 antibodies (lower panels) and detection of ADAM15 or NCAD with the respective antibodies. (**B**) Analogous to (**A**) Co-IPs using NCAD and ADAM15 antibodies in chondrocyte cell lines transfected with full length ADAM15 plasmid (+) or with ADAM15 lacking the cytoplasmic tail (Δcyto) and an empty vector (-). Mouse IgG served as a control. Input: 10% total lysate. (**C**) confocal microscopy of RASFs double immunofluorescently stained for NCAD (green) and ADAM15 (red). White inset indicates area of the magnified merged image. (**D**) In situ proximity ligation assays for ADAM15 and NCAD, showing direct interaction of both molecules in densely seeded cells only (arrows). Cell nuclei were counterstained with DAPI. Objective 40x, scale bar = 10 μm.

To analyze, whether ADAM15 interacts with N-cadherin in adherens junctions in situ, co-immunoprecipitations (IPs), double immunofluorescence stainings and proximity ligation assays were employed. IPs using anti-NCAD antibodies in RASFs with prior silencing of ADAM15 revealed binding of NCAD to ADAM15 in ADAM15-expressing cells only, and vice versa using anti-ADAM15 antibodies detected binding of ADAM15 to NCAD (Fig. 3A). Analogously, IPs of NCAD and ADAM15 in lysates from chondrocyte cell lines transfected with full length ADAM15 (~ 100 kDa) and with ADAM15 lacking the cytoplasmic tail (Δcyto) (~ 90 kDa) revealed binding of NCAD to both ADAM15 constructs (Fig. 3B), thus showing that the extracellular domains of ADAM15 are responsible for NCAD binding.

Double immunofluorescence staining of RASFs for NCAD and ADAM15 showed co-localization of both molecules at the cell membrane (Fig. 3C). In addition, proximity ligation assays – a method whose in situ detection of 2 proteins in close proximity requires their colocalization within low molecular distance dimensions (< 40 nm) – exhibited colocalized ADAM15 and NCAD at the cell membrane of densely seeded compared to single cells (Fig. 3D), further corroborating the direct interaction of both proteins in cells forming adherens junctions.

### Mechano-induced recruitment of PAK2 and Nck to the cell membrane

**Fig. 4:**
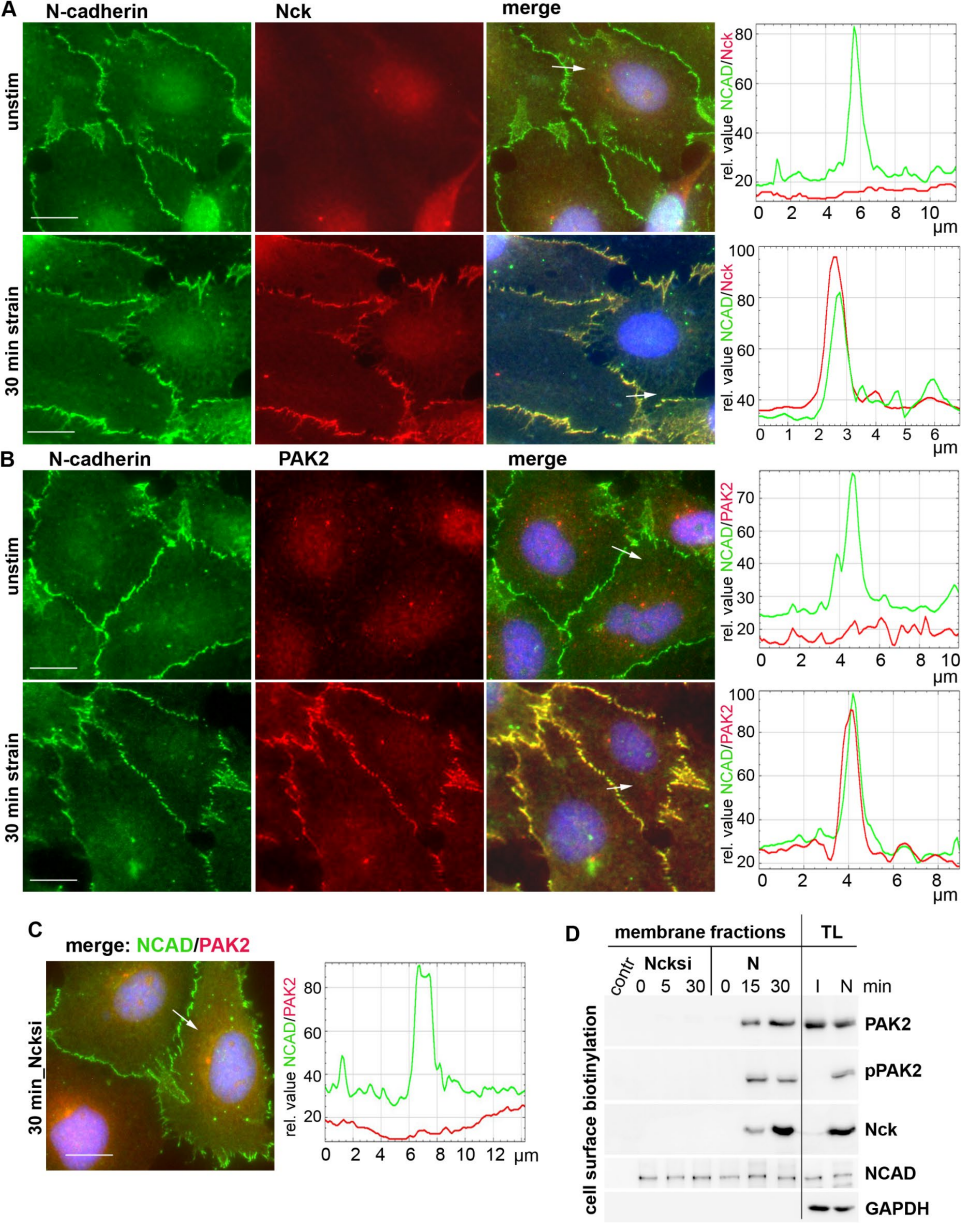
Mechano-induced recruitment of PAK2 together with Nck to the cell membrane. (**A**,** B**) RASFs grown on Bioflex plates and either unstimulated or strained for 30 min were fluorescently stained for (**A**) NCAD and Nck and (**B**) NCAD and PAK2. Adjacent to images: quantification of the pixel density of NCAD /Nck or NCAD /PAK2 along the white arrow. (**C)** double staining of NCAD and PAK2 in RASFs with prior silencing of Nck, right, pixel density along arrow. Cell nuclei were counterstained with DAPI. Objective 40x, scale bar = 10 μm. (**D**) immunoblots for PAK2, pPAK2, Nck and NCAD. RASFs pre-treated with Nck siRNA (I) and a nonsilencing siRNA (N) were mechanically strained for 15 and 30 min, the cell surface biotinylated with non-membrane-permeable biotin reagent and purified on streptavidin-conjugated magnetic beads. Contr: non biotinylated cell lysates enriched on streptavidin beads served as background control. TL = total lysates from 30 min time point. GAPDH was used to control the purity of the plasma membrane fractions.

Next, we analyzed, whether mechano-induced PAK2 activation is accompanied by the recruitment of PAK2 and its binding partner SH2/SH3 adapter protein Nck1^20^, which links PAKs with cell membrane-anchored receptor tyrosine kinases^21^, to the cell membrane. Localization of pPAK/PAK2 and Nck was analyzed by double immunofluorescence stainings with NCAD. In unstimulated RASFs staining of NCAD was detected at the cell membrane and a homogenous cytoplasmic localization of Nck and PAK2 was detected (Fig. 4A, B). However, upon mechanical stimulation Nck, PAK2 and p-PAK2 (see Supplementary Fig. 1) were detected at the cell membrane in co-localization with NCAD, clearly indicating that both molecules redistribute from the cytoplasm to the cell membrane upon induction of mechanical strain.

To analyze, whether Nck indeed impacts the redistribution of PAK2 to the cell membrane, PAK2 staining in cells and purified cell membrane fractions upon Nck knockdown were performed. Silencing of Nck did not result in any redistribution of PAK2 to the cell surface upon strain induction, as shown by immunostaining (Fig. 4C). Accordingly, cell surface biotinylation and enrichment of membrane fractions on streptavidin-conjugated magnetic beads revealed that detectability of PAK2 and phosphorylated PAK2 in membrane fractions is limited to Nck-expressing cells, as silencing of Nck completely blocked recruitment of PAK2 to the membrane (Fig. 4D), thus clearly showing that Nck is crucially required for the redistribution of PAK2 to the cell surface triggered by mechanical strain.

### Binding of PAK2 to ADAM15/NCAD complex at cell membrane is crucially dependent on ADAM15

**Fig. 5:**
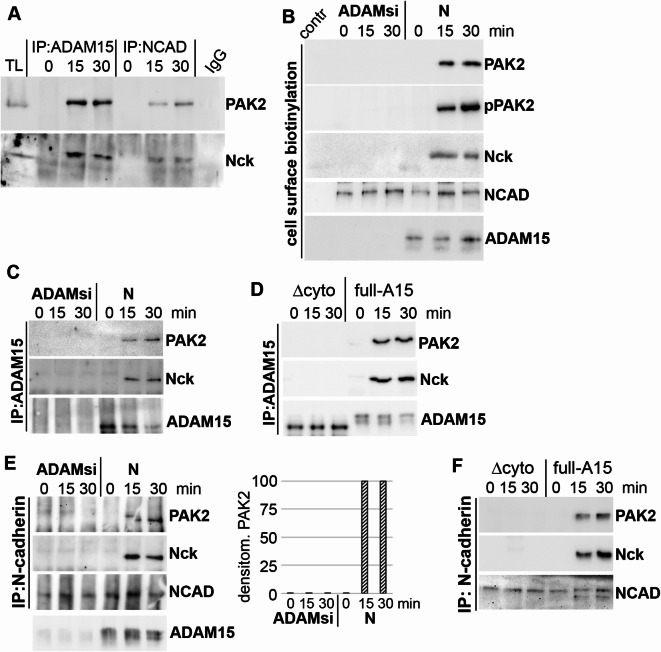
Binding of PAK2 to ADAM15/NCAD complex at cell membrane is crucially dependent on ADAM15. (**A**) IPs of strained RASFs using ADAM15 or NCAD antibodies and detection of PAK2 and Nck. TL = total lysate. IgG, mouse IgG served as background control. (**B**) cell surface biotinylation and enrichment of membrane fractions on streptavidin beads of strained cells with ADAM15-silenced or nonsilenced (N). Contr: non biotinylated cell lysates enriched on streptavidin beads served as background control. (**C**,** D**) IPs using ADAM15 antibodies in (**C**) RASFs with silenced and nonsilenced ADAM15 expression (N) and (**D**) in chondrocyte cell lines transfected with full length ADAM15 (full-A15) or ADAM15 lacking the cytoplasmic domain (Δcyto). (**E**,** F**) analogous to (**C**,** D**) IPs using N-cadherin antibodies, showing precipitates of PAK2 and Nck in ADAM15-expressing cells only. (**E**) right, densitometric evaluation of PAK2 from experiments obtained from 4 donors.

Next, we analyzed, whether mechano-induced Nck/PAK2 redistribution to the cell membrane is dependent on NCAD and/or ADAM15 expression using co-immunoprecipitations (IP) of either total cell lysates or purified membrane fractions derived from surface biotinylated RASFs. IPs of total cell lysates using ADAM15 antibodies resulted in precipitation of Nck and PAK2 exclusively in mechanically strained but not in unstimulated RASFs (Fig. 5A). The amount of precipitated Nck and PAK2 from strained cells remained reduced when using NCAD antibodies for precipitation, suggesting their primary binding to ADAM15 rather than to NCAD. These results were confirmed by immunoblot analysis of cell surface biotinylated and streptavidin-purified membrane fractions of RASFs with ADAM15-silenced or nonsilenced expression (Fig. 5B). Additionally, IPs using ADAM15 antibodies showed precipitated Nck/PAK2 in ADAM15-expressing RASFs and in chondrocyte cell lines transfected with full length ADAM15 only in mechanically strained fractions (Fig. 5C, D). However, in ADAM15-silenced as well as cells transfected with an ADAM15 deletion mutant (Δcyto) lacking the cytoplasmic domain, Nck/PAK2 could not be precipitated anymore, showing that the cytoplasmic domain of ADAM15 is indispensable for the interaction with Nck and/or PAK.

In parallel IP-experiments using NCAD antibodies, Nck and PAK2 could be precipitated in all lysates from strained cells expressing ADAM15 and/or full length ADAM15 (Fig. 5E, F). However, silencing of ADAM15 and/or the deletion of its cytoplasmic domain completely abrogated the precipitation of Nck/PAK2.

Together, these results show that mechanical strain triggers the redistribution of Nck and PAK2 from the cytoplasm to membrane-anchored ADAM15 and NCAD, and that the binding of Nck/PAK2 to the ADAM15/NCAD complex is crucially dependent on the expression of ADAM15.

### Cadherin-11 is a target of ADAM15/NCAD-downregulated H19

**Fig. 6:**
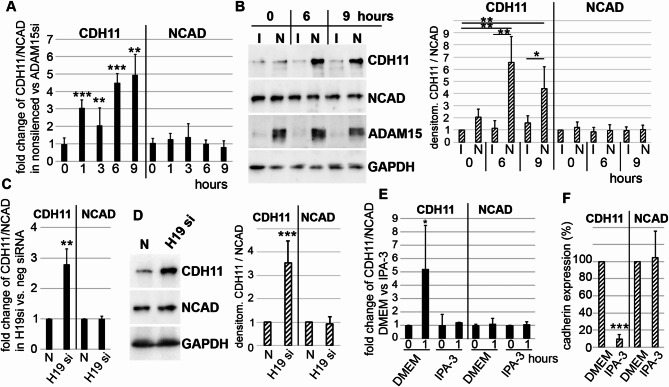
Cadherin-11, but not Cadherin-2 (NCAD) is a target of mechano-downregulated H19. (**A**,** B**) RASFs with ADAM15-nonsilenced (N) or silenced (I) were mechanically strained and (**A**) RT-qPCR, fold changes of CDH11 or NCAD from 5 different RASFs and (**B**) immunoblots for CDH11 and NCAD, right, densitometry for CDH11 and NCAD from 4 different RASFs. (**C**,** D**) unstimulated RASFs with H19 silenced versus nonsilenced, (**C**) RT-qPCR, fold changes of CDH11 and NCAD from 5 different RASFs, (**D**) immunoblots for CDH11 and NCAD, right, densitometry from 4 different RASFs. (**E**) RT-qPCR for CDH11 and NCAD from mechanically strained (1 h) RASFs incubated with PAK inhibitor IPA-3 or DMEM medium. (**F**) densitometric evaluation of immunoblots for CDH11 and NCAD from 4 different RASFs (mean ± SD) treated with DMEM or IPA-3 for 48 h. GAPDH served as loading control. **p < 0.005, ***p < 0.0005, Student’s t-test, comparing DMEM versus inhibitor.

Since our investigations have so far identified in RASFs a mechano-sensitive pathway of ADAM15/NCAD-dependent activation of the serine/threonine kinase PAK2, which could be identified as crucial to strain-induced downregulation of H19 in these cells, we focused on the elucidation of the effector arm of this regulatory circuit. It has been shown that H19 can regulate the expression of E- and N-cadherins^22^. Therefore, we examined whether N-cadherin and cadherin-11, expressed in synoviocytes from synovial membranes, might be candidate targets of ADAM15-mediated H19-dependent mechano-induced gene regulation. In ADAM15-expressing RASFs, significant increases of CDH11 mRNA (up to ~ 5 fold) and protein levels as compared to ADAM15 nonexpressing cells could be detected upon mechanical strain (Fig. 6A, B), whereas NCAD mRNA and protein levels remained unaffected. Additionally, silencing of NCAD resulted in the mechano-induced downregulation of H19 and concomitant selective upregulation of CDH11 (see Supplementary Fig. 2), further supporting the requirement of the interaction of NCAD and ADAM15 in the H19 downregulation and subsequent CDH11 upregulation.

Also, H19 silencing alone without any stimulus resulted in a significant ~ 3 fold upregulation of CDH11 mRNA and protein levels, but not of NCAD (Fig. 6C, D), thus supporting the contribution of mechano-induced H19 downregulation in ADAM15/NCAD-mediated increased CDH11 expression.

Next, we analyzed, whether inhibition of PAK signaling, which inhibited the mechano-induced downregulation of H19, also affects CDH11 expression. Inhibition of PAKs using IPA-3 resulted in the inhibition of mechano-induced CDH11 mRNA, but not NCAD mRNA levels, and also protein expression levels (Fig. 6E, F), clearly showing that PAK signaling plays a pivotal role in the H19 and CDH11 regulation.

### Identification of miR-130a-3p regulating CDH11 expression

**Fig. 7:**
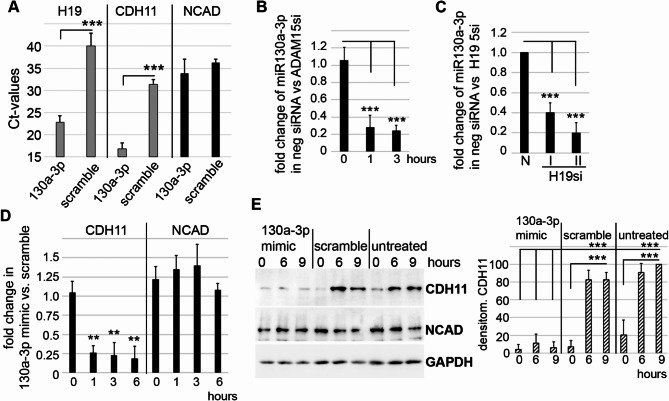
ADAM15/H19-dependent downregulation of miR-130a-3p, which binds to 3’ UTR of cadherin-11 and regulates its expression. (**A**) RNA pull-downs using transfected miR-130a-3p or scrambled mimics, purification on streptavidin beads and detection of binding using qPCR of H19, CDH11 and NCAD, showing Ct values of 5 different RASFs (mean ± SD). (**B**) RT-qPCR of miR-130a-3p in ADAM15-expressing or silenced RASFs, showing the mean ± SD of fold changes from 5 different RASFs. (**C**) RT-qPCR for miR-130a-3p in RASFs silenced with two different H19 siRNAs (I and II) vs. negative control siRNA (N). (**D**,** E**) RASFs prior transfected with 130a-3p mimics or scramble were strained and (**D**) RT-qPCR for CDH11 and NCAD and (**E**) immunoblots for CDH11 and NCAD; untreated = strained without any treatment; right panel, densitometry for CDH11 from 4 different RASFs. GAPDH served as loading control. **p < 0.005, ***p < 0.0005, Student’s t-test.

Since lncRNAs are known for their potential to affect gene regulation via interaction with miRNAs, we performed an in silico analysis of RA-associated candidate miRNA binding sites in H19. For the candidate miR-130a-3p^14^, our data base search (https://diana.e-ce.uth.gr/lncbasev3) revealed prediction of binding sites in H19. To validate miR-130a-3p binding to H19, RNA pull-downs using lysates from 5 different RASFs prior transfected with biotinylated miR-130a-3p or scrambled mimics, purification on streptavidin beads and subsequent qPCR were performed. A binding of 130a-3p mimics to H19 was detectable, as shown by low Ct levels, but virtually no binding (≥ 40 Ct) of the scrambled mimic to H19 was detected (Fig. 7A), thereby experimentally confirming in vitro binding of miR-130a-3p to H19.

In parallel, significant binding of miR-130a-3p mimic to CDH11 RNA was detected compared to the scramble control, whereas no binding of miR-130a-3p (≥ 35 Ct) to NCAD was detectable (Fig. 7A), confirming the selective binding of miR-130a-3p to CDH11 but not NCAD mRNA.

To analyze, whether miR-130a-3p is regulated by mechanical strain in dependency on ADAM15, RASFs with prior silencing of ADAM15 or silenced with a negative control siRNA were strained and RT-qPCR for mature miR-130a-3p performed. In ADAM15-expressing RASFs, miR-130a-3p was mechano-downregulated by 4-5x fold compared to ADAM15-silenced cells (Fig. 7B). To analyze, whether the observed miR-130a-3p mechano-downregulation is due to concomitant H19 downregulation, H19 was silenced using 2 different siRNAs and a nonsilencing siRNA and mir-130a-3p levels determined by RT-qPCR. H19 silencing alone without applying strain resulted in a significant 4–5 fold downregulation of miR-130a-3p compared to RASFs with nonsilenced H19 (Fig. 7C), which might indicate that H19 sponging miR-130a protects it from degradation.

To analyze, whether miR-130a-3p indeed influences mechano-induced CDH11 expression, RASFs prior transfected with miR-130a-3p or scrambled mimics were strained and CDH11 and NCAD mRNA and protein levels determined by qPCR and immunoblotting. Contrary to the expression levels of NCAD, both CDH11 mRNA and protein levels were significantly reduced in cells transfected with miR-130a-3p mimics compared to scramble controls (Fig. 7D, E).

Moreover, in parallel experiments, the same influence of miR-130a-5p mimics on CDH11 mRNA and protein level is shown (see Supplementary Fig. 3). Potential binding sites of miR-130a-3p and miR-130a-5p to the 3’ UTR of CDH11 are shown in Supplementary Fig. 4. Together, these results clearly show that mechanical strain is able to trigger H19/miR-130a-mediated CDH11 upregulation.

### **Mechano-upregulated CDH11 significantly increases cell invasive behavior of RASFs**

**Fig. 8:**
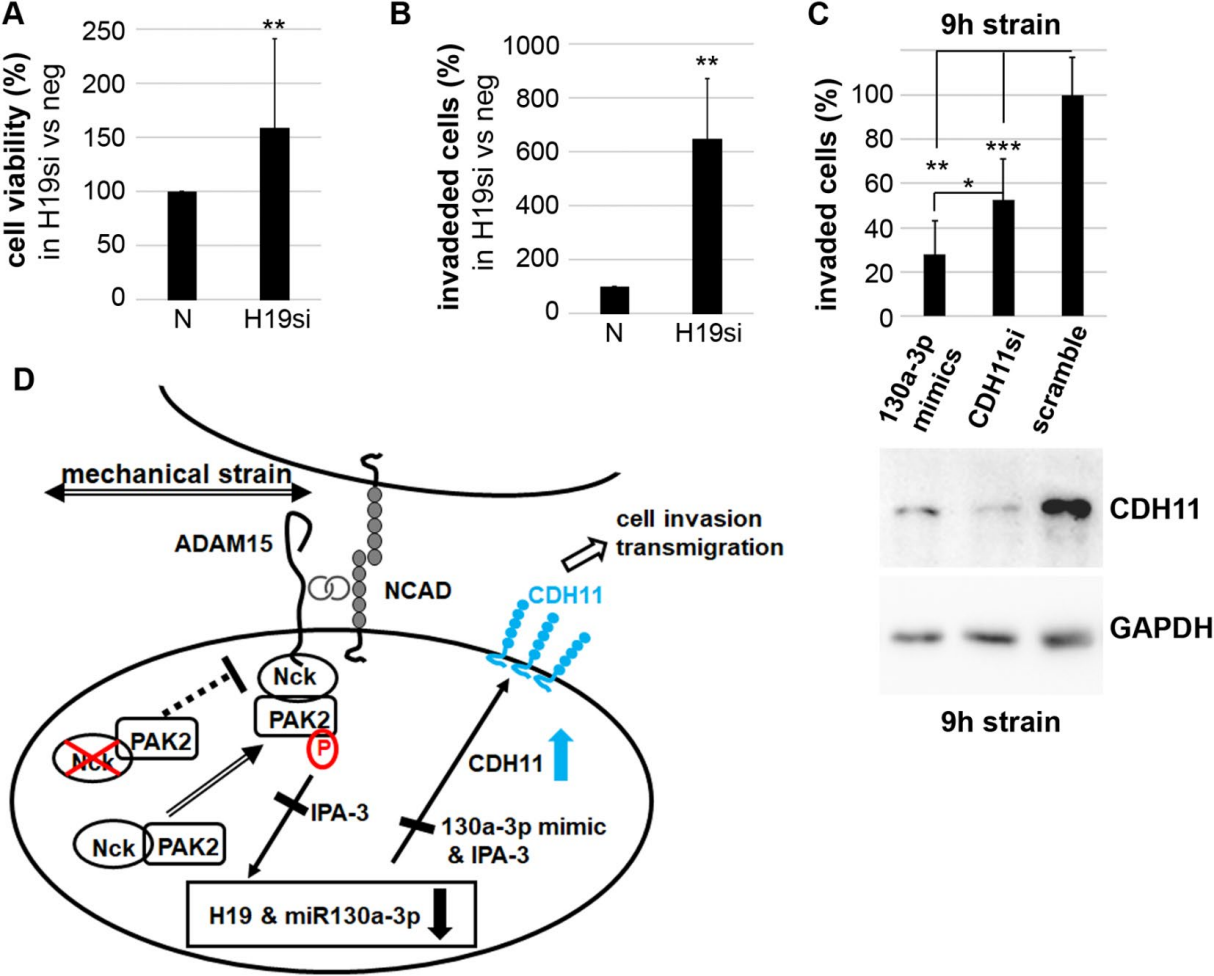
CDH11-mediated increased cell invasion can be blocked by CDH11 silencing and transfection with miR-130a-3p mimics. (**A**,** B**) RASFs were treated with H19 siRNA or negative nonsilencing siRNA (N) and (**A**) MTT-based cell viability assays and (**B**) cells transmigrated through matrigel were stained with DAPI, the whole transwell photographed and all cells counted. (**C**) upper panel, invaded cells prior transfected with 130a-3p mimics, CDH11 siRNA or scramble control, shown is the mean ± SD from 5 different RASFs. (**C**) lower panel, immunoblots for CDH11 from cells transfected and strained as in upper panel. *p < 0.05, **p < 0.005, ***p < 0.0005, Student’s test. (**D**) diagram of summarized results: mechanical strain results in NCAD/ADAM15-mediated phosphorylation of PAK2, resulting in the downregulation of lncRNA H19 and miR-130a-3p and subsequent upregulation of CDH11, which in turn promotes an aggressive phenotype: increased cell invasion. Inhibition of PAK signaling by PAK inhibitor IPA-3 blocks mechano-induced H19 downregulation and subsequently CDH11 upregulation. In addition, mechano-induced PAK2 phosphorylation is accompanied by recruitment of SH2/SH3 adapter Nck and PAK2 to the NCAD/ADAM15 complex, but binding of Nck/PAK2 is restricted to the cytoplasmic domain of ADAM15. The mechano-induced redistribution of PAK2 to the cell membrane does not occur when Nck is silenced.

To analyze, whether H19 and/or H19-mediated upregulated CDH11 influences cell viability or migration and matrix invasion, cell functional assays were performed. RASFs with silenced H19 showed significantly enhanced cell viability in a MTT-based viability assay compared to nonsilenced cells (Fig. 8A). Also, H19-silenced RASFs displayed a significantly increased transmigratory capacity through matrigel compared to nonsilenced cells (Fig. 8B), clearly showing an influence of H19 on cell viability as well as cell migration and matrix invasion.

Next, we investigated whether the H19 target CDH11 affects the invasive cell behavior of mechanically stressed RASFs. For this purpose, CDH11 was knocked down with siRNA or transfected with 130a-3p mimics before exposing the cells to mechanical stress for 9 h and finally measuring the cell infiltrative potential in matrigel. Significantly higher numbers of RASFs transfected with scramble control invaded into the matrigel as compared to RASFs transfected with either CDH11 siRNAs or 130a-3p mimics (Fig. 8C). This altered invasive behavior corresponds to the specific downregulation of CDH11 by siRNAs and to a higher degree by 130a-3p mimics, as shown in the immunoblots (Fig. 8C, lower panel), clearly showing the importance of CDH11 upregulation for the induction of pro-invasive properties in RASF by mechanical stimuli. A summary of all results from the present study is shown in Fig. 8D.

## Discussion

Joint damage caused by mesenchymal cells of the synovial lining during immune-mediated inflammation in rheumatoid arthritis preferentially affects site-specific mechano-sensitive areas, as revealed e.g. by an MRI investigation of erosive hand synovitis in early RA. The predilection of erosions to periligamentous locations at the radial sites of the 2nd and 3rd metacarpophalangeal joints^23^ could be predicted by known anatomic and biomechanical factors pertaining to the radial collateral ligaments at these sites^24^. In the present study, we have uncovered a new innate immune mechanism in RASFs likely contributing to strain-induced tissue damage at such mechano-sensitive sites of RA joints. Central to the elucidated pathway is the mechano-induced downregulation of the lncRNA H19 resulting in reinforced cell viability, as well as increased cell mobility and infiltrative growth mediated via upregulation of CDH11, an adherens junction molecule associated with an invasive cellular phenotype^17^. The upstream events leading to the transmission of the mechanical signals into downregulated H19 turned out as being dependent on ADAM15. This is in line with our previous studies on mechano-regulated lncRNAs, which had uncovered only 2 (Hotair and H19) out of 372 investigated disease-relevant lncRNAs as being downregulated in ADAM15-dependency in strained synovial fibroblasts^18^. As the expression of lncRNA Hotair was shown to be regulated by JNK kinase signaling and upstream Ca^2+^-signaling effectors, e.g. CaMKs and the mechano-sensitive Ca^2+^-channel TRPV4, our initial investigations focussed on the question, whether mechano-regulation of H19 depends on the same and/or different signaling pathways. However, inhibition of Ca^2+^-signaling effectors, JNKs and the Src family tyrosine kinases did not affect H19 expression contrary to their earlier described role in mechano-regulation of lncRNA Hotair^18^. Also, despite the well documented role of Src in ADAM15-mediated signaling via direct binding^6,25^, mechanically modulated H19 levels remained unaffected by blocking this tyrosine kinase with the dual Src/Abl inhibitor dasatinib. However, the complete blockade of mechano-induced H19 downregulation following the inhibition of PAK signaling by IPA-3 in the current study demonstrates the critical importance of this pathway for H19-mediated mechanotransduction.

Moreover, the finding that mechano-sensitive activation of PAK2 in the strained RASFs is strictly dependent on cell density indicates a crucial involvement of mechanotransduction mechanisms initiated at the adherens junctions. Mechanical strain is not only perceived by integrins, sensing mechanical clues from the extracellular matrix^26^, but also via adherens junction-associated cadherins, which sense, transmit and initiate adaptive cellular responses to mechanical forces imposed by neighbouring cells^27^. Compared to chondrocytes in cartilage, which exist as single cells embedded in the extracellular matrix and perceive mechanical load by integrins^28^, the mechanical forces in the joint during movement are sensed in the inflamed synovial membrane. This synovial tissue lines the inner surface of synovial joint capsules. In inflammatory conditons the synovial lining layer, which is composed of 1–2 cell layers in a healthy joint, becomes hyperplastic, a feature describing a highly hyperproliferative, densely packed tissue containing multi-layered fibroblasts. Thus, the detection of mechano-induced PAK2 activation in strained synovial fibroblasts seeded at high cell density in vitro mimics mechanical force perception by the synoviocytes in the hyperplastic synovial tissue of inflamed joints.

The newly uncovered mechano-induced activation of PAK2 in RASF clearly depended on both NCAD and ADAM15. This was revealed by knockdown of either of both molecules, whose direct interaction in adherens junctions of RASFs was demonstrated by immunoprecipitations and immunostainings using proximity ligation in situ. The disintegrin metalloproteinase ADAM15, capable of binding to integrins via its disintegrin domain in vitro^29^, is highly expressed in fibroblasts of RA synovial tissue in situ^3,30^. Despite its in vitro detected binding to integrins, ADAM15 was also detected in adherens junctions of endothelial cells preferentially colocalizing with VE-cadherin but in poor correlation to integrin β1 expression^19^. Triggering of PAK2 activation by mechanical forces has been described previously, namely by force on E-cadherin in human epithelial cells^31^, by shear stress on endothelial cells leading to enhanced proinflammatory NF-κB activation^32^ and by contraction stimuli of skeletal muscle cells with regulatory effects on glucose transport^33^. Besides mechanosensitive recruitment of PAK2, the SH2/SH3 domain-containing adaptor protein Nck proved crucial for upstream activation of mechano-induced ADAM15/NCAD–dependent PAK2 activation, as evidenced by the blocking effect of Nck knockdown. Accordingly, it has been shown in 293T fibroblasts^34^ and murine macrophages^35^ that the signaling activity of PAK requires its localization at the cell membrane via Nck-mediated recruitment. In analogy, the interaction of PAK1 with Nck is well documented, and upon stimulation Nck is recruited together with PAK1 to growth factor receptors via its SH2 domain^20,21^, thereby linking Nck/PAK signaling with cell membrane receptors.

Here, we uncovered that ADAM15 expression is crucial for the recruitment of Nck and PAK2 to the ADAM15/NCAD complex. Nck binds to the cytoplasmic domain of ADAM15 upon induction of mechanical strain and the deletion of this domain results in a complete abrogation of Nck/Pak2 redistribution to the cell membrane. Accordingly, the cytoplasmic domain of ADAM15 is known to interact in vitro with SH2/SH3 domain containing proteins, including Src and Nck^4,5^. In addition to the necessity of cell membrane recruitment of PAK2 for its activation^34^, an involvement of this kinase in cadherin-mediated mechanosensing was previously demonstrated for E-cadherin in epithelial cells^31,36^. In this respect, our investigation provides first evidence that the complex consisting of N-cadherin and ADAM15, expressed by mesenchymal synoviocytes and formed via interactions of their extracellular domains in adherens junctions, is required for mechanosensing via Nck-mediated PAK2 recruitment and its subsequent activation via binding to the cytoplasmic domain of ADAM15. Moreover, our study uncovers for the efferent loop of this mechanosensing pathway in RASFs that PAK2 activation finally results in downregulation of H19 associated with a significant increase of CDH11 expression. Although only sparse information on mechano-regulation of lncRNA H19 is available, H19 has been described as a mechano-sensitive lncRNA acting as a positive regulator of mechanical tension-induced osteogenic differentiation of bone marrow mesenchymal stem cells^37^.

Our studies do not only demonstrate the association of mechanically downregulated H19 with an increased expression of cadherin11 as a pro-invasive factor, but also experimentally prove the causal relationship between the downregulation of H19 with upregulation of CDH11 and invasive cell behavior. Thus, we could show that H19 silencing of resting RASFs alone causes upregulation of CDH11, thereby increasing significantly their transmigratory capacity through matrigel that was blocked by CDH11-specific siRNA as well as miR-130a-3p mimics targeting CDH11. In this respect, our data in RASFs differ from reported studies of tumor cell lines in which high levels of H19 could be linked to oncogenic properties such as epithelial-mesenchymal transition (EMT) associated with invasive, migratory and metastatic properties^38,39^. However, the hallmark of EMT is the cadherin switch characterized by the upregulation of N-cadherin and downregulation of E-cadherin. In contrast to epithelial tumor cells, primary fibroblasts of RA synovial tissue do not express epithelial E-cadherin but N-cadherin and CDH11 due to their mesenchymal phenotype. CDH11 expression in malignancies e.g. breast cancer is associated with poor outcome and has been also reported to promote a pro-invasive behavior in synovial fibroblasts^40–42^, rendering CDH11 an attractive target for therapeutic intervention^43^.

Based on the known interactions of lncRNAs with miRNAs in gene regulation, an in silico analysis of candidate miRNA-binding sites in H19 was performed, which unexpectedly revealed a previously unknown antagonist of CDH11 upregulation in RASFs. Our database screen, which focused on miRNAs previously described in the context of RA, identified miR-130a-3p as a candidate, which targets both H19 and CDH11 RNA and was downregulated in the present study under mechanical stress in dependency on ADAM15 expression. However, the decrease of miR-130a-3p with concomitant upregulation of CDH11 expression was not restricted to force-induced H19 modulation, but also occurred upon H19 silencing by specific siRNAs in RASFs not subjected to mechanical strain. This indicates a direct causal relationship between H19 downregulation and reduced miRNA130a-3p levels. However, the underlying molecular mechanism of action finally leading to CDH11 upregulation is less clear. A sponging adsorption effect based on the simple binding of miR130a-3p directed against CDH11 to lncRNA H19 is not sufficient as an explanation, since in this case downregulation of H19 should lead to a reverse increase in miR130a-3p levels and subsequent downregulation of CDH11, which disagrees with the experimental evidence of the present study. Whereas the exact mechanism of H19 and miR-130a-3p interplay in a complex network of ceRNAs (competing endogenous RNAs) is still elusive, our results on the downregulation of miR-130a-3p by H19 silencing are in line with earlier studies of the interaction of these RNA species in the modification of radio- and chemo-sensitivity of neoplastic cardiac cells^44^.

During immune-mediated chronic joint inflammation in RA, fibroblasts residing in the hyperplastic synovial membrane constantly sense mechanical forces transmitted during joint motion and develop an aggressive phenotype characterized by proteolytic attacks on the extracellular matrix (ECM) and infiltrative growth into cartilage and bone making them attractive targets for drug development^2,45^. In this respect, our studies have uncovered a new and crucial mechano-inflammatory pathway in RASFs. It is triggered by the perception of mechanical forces at the adherens junctions of densely populated RASFs leading to H19 and miR-130a-3p dependent upregulation of CDH11 associated with a shift towards a pro-invasive cellular behaviour. Accordingly, immunohistochemical studies have revealed an abundant detection of cadherin-11 expression in RASFs of metacarpophalangeal joints localized in typically biomechnical stressed sites at the junction between cartilage and aggressively growing synovial tissue^40^. Thus, the newly elucidated pathway likely contributes to mechanosensitive site-specific variability of the aggressive phenotype of RASFs in RA-pathogenesis and may open new avenues for therapeutic intervention.

## Methods

### Cell culture

Synovial fibroblasts were isolated from tissue obtained during joint replacement/arthroscopic synovectomy at the Clinic of Orthopedics, University Hospital Jena, Germany. All patients had provided written informed consent and approval was obtained from the Ethics Committee of the Jena University Hospital. Also, methods were performed in accordance with the relevant guidelines and regulations of the committee. Synovial fibroblasts were isolated from hip or knee joints, as described earlier^46^. Briefly: Tissue samples were minced and digested with trypsin/collagenase P. The resulting single cell suspension was cultured for 1 week and non-adherent cells removed by medium exchange. Then, CD14 + monocytes/macrophages were removed using Dynabeads coated with anti-CD14 antibodies, RASFs cultured over 2 passages in DMEM, containing 10% FCS and penicillin/streptomycin (100 µg/ml), and cells between passages 3–6 used. In total, 6 different RA synovial fibroblast lines from 6 different donors were studied and the exact number of cell lines used for experiments indicated in figure legends. The chondrocyte cell line T/C28a4 transfected with full-length ADAM15, the deletion mutant without the cytoplasmic domain, or vector control were generated and grown in DMEM/10% FCS, as described previously^47^.

### Cyclic tensile strain

For application of strain the Flexcell FX-3000 System (Flexcell International Corp, Hillsborough, USA) was used. This computer-based system uses controled vacuum to strain cells adhered to flexible rubber membranes. Vacuum is applied to a loading station, into which four 6-well culture plates are mounted. RASFs (3.5 × 10^5^ cells/6-well) were grown in BioFlex^®^ collagen type I culture plates for 2–3 days and subjected to continuous mechanical stimulation with an elongation of 15% and a frequency of 1 Hz for various time points at 37° C in 5% CO_2_. Unstimulated cultures were grown under the same conditions but without the strain protocol.

### Inhibitor assays

RASFs (3.5 × 10^5^ cells/6-well) grown in BioFlex/type I collagen plates were pre-incubated for 30 min with DMEM containing inhibitors and subjected to strain for various time points using SP600125 (50 µM), dasatinib (1 µM), KN-93 (50 µM), IPA-3 (50 µM) from Tocris Bioscience. Trifluoperazine (TFP; 50 µM) from Santa Cruz Biotechnology.

### Preparation of cell lysates and western blotting

After the stimulation, RASFs were washed with ice-cold PBS, scraped off and lysed in RIPA buffer (50 mM Tris, pH 7.0, 150 mM NaCl, 5mM EDTA, 1% Triton X-100, 0.25% sodium deoxycholate and complete proteinase inhibitor cocktail (Roche Diagnostics) and phosphatase inhibitor cocktail I and II (Roche Diagnostics, 10 µl/ml lysis buffer). Samples were applied in loading buffer (6x stock solution: 375 mM Tris-HCl, 9% SDS (w/v), 50% glycerol (v/v), 0.03% bromophenol blue (w/v), 9% β-mercaptoethanol (v/v)), separated by 10% SDS/PAGE, transferred to nitrocellulose filter, and blocked with 5% milk powder (Roth) in PBST (137 mM NaCl, 2.7 mM KCl, 10 mM Na_2_HPO_4_, and 1.8 mM KH_2_PO_4_, 0,1% Tween20, pH 7.4) for 30 min at room temperature and incubated with antibodies diluted in 1% milk powder/PBST against phospho-PAK2 (Ser20) (Cell Signaling Technology, #2607, dilution 1:1000), phospho-PAK1 (Ser144)/PAK2 (Ser141) (Cell Signaling Technology, #2606, 1:1000), Nck1 (Cell Signaling Technology, #2319, 1:1000), PAK2 (R&D Systems, #MAB6849; 1:1000), ADAM15 (R&D Systems, #AF935, 1:1000), OB-Cadherin/Cadherin-11 (Santa Cruz Biotechnology, #sc-365867; 1:1000), N-cadherin (R&D Systems, #AF6426; 1:1000), GAPDH (Santa Cruz Biotechnology, #sc-365062; 1:10.000) overnight at 4 °C and developed using appropriate horseradish peroxidase-conjugated antibodies (Cell Signaling Technology, horse anti-mouse IgG #7076, horse anti-rabbit IgG #7074 (1:2500 each), donkey anti-Goat IgG (Thermo Fisher #A15999; 1:2500). For detection of immunoprecipitates HRP-conjugated secondary antibody recognizing only the native primary antibody (VeriBlot for IP Detection Reagent, Abcam) was used. Signals obtained with chemiluminescence reader Fusion FX (Vilber, France) were evaluated using ImageJ.

### Co-immunoprecipitation

RASFs (3.5 × 10^5^/6-well) or T/C28a4 chondrocyte cells (5 × 10^5^/6-well) grown in BioFlex plates for 48 h were washed with ice-cold PBS, and lysed in a lysis buffer (10 mM HEPES, pH 7.0, 150 mM NaCl, 1% Triton X-100, complete proteinase inhibitor cocktail (Roche Diagnostics; 10 µl/ml lysis buffer) for 1 h at 4 °C. Lysates from 3 pooled 6-wells (200 µg) were incubated with ADAM15 (R&D Systems, #AF935) or N-cadherin (R&D Systems, #AF6426) antibodies (1 µg/mg lysate, respectively) and 25 µl protein G-conjugated agarose beads (Santa Cruz) under constant agitation overnight at 4 °C. Beads were washed 4 times with lysis buffer, subjected to SDS/PAGE, and Western blotting, as described above.

### Transfection using SiRNA and MiR mimics

To trypsinized synovial fibroblasts (3.5 × 10^5^ cells/500 µl DMEM/10% FCS) 55 µl transfection mixture containing 5 µl Saint-sRNA transfection reagent (Synvolux, Leiden, NL) and the respective siRNA (20 nM, Silencer Select predesigned and validated small interfering RNAs (Thermo Fisher) or miR mimic (50 nM, Qiagen) was added. Cells were seeded into a 6-well, allowed to adhere for 1 hour, volume adjusted to 2.5 ml with DMEM/10% FCS and incubated for 48 h. siRNAs used: ADAM15 (ID: n16681) 5’-GAUCUACUCUGGGAGACAA-3’, H19 I (ID: n272453) 5’-UCAUCAGCCCAACAUCAA-3’, H19 II (ID: n502892) 5’-CUCCCAGAACCCA CAACA-3’, NCAD siRNA (I) (ID: s2772) 5’-GGGUAAUCCUCCCAAAUCA-3’, NCAD siRNA (II) (ID: s2771) 5’-GUGCAACAGUAUACGUUAA-3’, cadherin-11 (ID: s2800) 5’-GAAUCCUGAUGGU AUCAAU-3’, Nck1 (ID: s9309) 5’-CCUCCACAGUGUGAUUA-3’, PAK2 (ID: s10024) 5’-CAGAGGUGGUUACACGGAA-3’, or a non-silencing siRNA #1 (#430843). miRCURY LNA miRNA were from Qiagen: miR-130a-3p mimic (YM00472237; 5’CAGUGCAAUGUUAAAAGGGCAU 3’), scramble (YM00479902; 5’UCACCGGGUGUAAAUCAGCUUG 3’).

### RNA pulldown assay

In order to validate mRNA targets for miR-130a-3p, the protocol from Dash et al.^48^ was used. Briefly: RASFs (3.5 × 10^5^) were transfected using 50 nM biotin-labeled miR130a-3p mimic (YM00472237-BDI, QIAGEN) or biotin-labeled scramble control (YM00479902-BDI) as described above. After 48 h cells were harvested and lysed in 150 mM NaCl, 25 mM Tris-Cl, pH 7.5, 5mM DTT, 0.5% IGEPAL, 60 U/ml Superase, protease inhibitor. Lysates were incubated with pre-washed streptavidin-coated magnetic beads (15 µl; Thermo Fisher) for 1 h at 4 °C and RNA isolated using the NucleoSpin RNA isolation kit (Macherey-Nagel) according to the supplier’s instructions. RNA was reverse transcribed using MLV reverse transcriptase (Promega) and analyzed by qPCR for H19, CDH11 and NCAD, as described under RT-qPCR.

### Semi-quantitative qPCR

RASFs (3.5 × 10^5^ cells/6-well) grown in BioFlex/type I collagen plates were subjected to mechanical strain for various time points. Total RNA was isolated using the RNeasy kit from Qiagen, according to the manufacturer’s instructions, and DNase I–treated RNA (500 ng) was reverse transcribed using M-MLV RT (Promega) and oligo d(T) primers. cDNA was amplified using SYBR^®^ Green Master Mix (Promega) on the QuantStudio™5 Real-Time PCR System (Applied Biosystems). Ct values were normalized to GAPDH and fold changes calculated using the 2^−∆∆Ct^ method. Primers used: H19 forward: 5’ GTGGACTTGGTGACGCTGTA 3’, H19 reverse: 5’ CACCATCCTCCCTCCTGAGA 3’, cadherin-11 forward: 5′ GATCGTCACTGACCTCGCA 3′, cadherin-11 reverse: 5′ CTTTGGCTTCCTGATGCCGATTG 3′, NCAD forward 5’ CATCATCATCCTGCTTATCCTTGT 3’, NCAD reverse 5’ GGTCTTCTTCTCCTCCACCTTCT 3’, GAPDH forward: 5’ GAAGGTGAAGGTCGGAGTC 3’, GAPDH reverse 5’ GAAGGTGAAGGTCGGAGTC 3’.

### RT-qPCR for mature miR-130a-3p

Cells were strained and RNA isolated as described above. For quantification of mature miR-130a-3p the miRCURY LNA miRNA PCR Starter Kit (Qiagen) was used. Briefly: During cDNA synthesis a poly(A) tail is added to the mature miRNA and cDNA synthesized using a universal poly(T) primer with a 3’ degenerate anchor and a 5’ universal tag. The cDNA template is then amplified using miR-130a-3p specific and LNA-enhanced forward and reverse primers (YP00204658) and SYBR^®^ Green for detection. Amplification of U6 snRNA was used for normalization.

### Isolation of cell surface biotinylated plasma membrane

RASFs (3.5 × 10^5^/6-well) were grown in Bioflex-plates for 48 h, washed twice with PBS and incubated with the membrane impermeable EZ-Link Sulfo-NHS-LC-LC-Biotin (0.1 mg/ml in PBS, pH 8.0; Pierce) for 15 min at room temperature. After washing twice with PBS, cells were fixed with 1% PFA in PBS for 5 min at room temperature, and quenched with 2.5 M glycine for 5 min. After harvesting, cell pellets were lysed in 10 mM HEPES, pH 7.0, 150 mM NaCl, 5mM EDTA, 1% Triton X-100, containing complete proteinase inhibitor cocktail (Roche Diagnostics) and ultrasonicated on ice. After removal of cell debris by centrifugation at 13.000 rpm for 5 min, the supernatant was incubated with streptavidin-conjugated magnetic beads (7.5 µl beads/3 pooled 6-wells) and 1U benzonase nuclease (Merck) under rotation for 2 h at room temperature. The magnetic beads were washed thrice with PBS/0.1% Triton X-100, and boiled in 20 mM Tris/HCl, pH 6.8, 0.5% SDS, 10% Glycerin v/v, 0,1% bromophenol blue w/v, 1% β-mercaptoethanol for 15 min at 99 °C to revert the PFA-crosslinked protein complexes.

### Immunofluorescence

RASFs (3.5 × 10^5^/6-well) were grown on Bioflex plates for 48 h, the rubber membrane thoroughly cleaned with cotton swabs to get rid of the vacuum grease, and ~ 1 cm^2^ slices cut out of the rubber membrane with a scalpell. Slices were glued with mounting medium to a glass slide and cells fixed with ice cold methanol for 5 min and aceton for 45 s. After blocking with 1% horse serum in PBS for 1 h, cells were incubated with sheep-anti N-cadherin (R&D Systems, #AF6426, 1:100) and rabbit anti-Nck (Cell Signaling Technology, #2319, 1:100), phospho-PAK1 (Ser144)/PAK2 (Ser141) (Cell Signaling Technology, #2606, 1:100), anti-phosphoPAK2 PAK2 (R&D Systems, #MAB6849; 1:50), antibodies overnight and visualized with Alexa Fluor 488 anti-goat and Alexa Fluor 594 anti-rabbit conjugated antibodies (1:500; Invitrogen). Images were acquired using EVOS FL Auto microscope (Thermo Fisher). For detection of ADAM15 and NCAD, cells (1 × 10^4^) were grown on 8-chamberslides for 48 h, fixed with 4% PFA, permeabilized with PBS/0.1% Triton X-100 for 5 min, blocked with 1% horse serum and incubated with mouse ADAM15 (R&D Systems, #MAB935, 1:50) and sheep-anti N-cadherin antibodies. Images were acquired using the Zeiss LSM 80 confocal laser scanning microscope. Nuclei were counterstained with mounting medium containing 4′,6-diamidino-2-phenylindole (DAPI). Digital images were processed and analyzed using ImageJ.

### Proximity ligation assays

For detection of direct interaction of ADAM15 and NCAD in situ, the NaveniFlex GM kit (Navenci, Sweden) was used according to the manufacturer’s protocol. Briefly: RASFs (1.5 × 10^4^) were grown on 8-chamber slide (Sarstedt) pre-coated with rat tail collagen type I (6 µg/cm^2^). After fixation with 4% PFA for 4 min, cells were permeabilized with PBS/0.1% Triton X-100 for 5 min and blocked with PBS/1% horse serum for 30 min. Cells were incubated with sheep N-cadherin (1:100) and mouse ADAM15 (1:50) overnight, then incubated with oligonucleotide-labeled secondary antibodies. Amplification of hybridized signal is performed using a polymerase mix containing fluorescent ATTO488 dye for 90 min at 37 °C. Only in case of close proximity of two proteins, the oligonucleotide-labeled secondary antibodies are able to hybridize, generating one single fluorescence signal.

### Cell viability assay

Cell viability was assessed using the RealTime-Glo™ MT cell viability assay (Promega) according to the manufacturer’s instructions. Briefly: After H19 silencing, RASFs were harvested by scraping and resuspended in 1 ml of DMEM. 50 µl of cell suspension was transferred to a white 96-well plate (Greiner), incubated with 50 µl of viability substrate for 1 h at 37 °C and luminescence measured using Mithras LB940 microplate reader. All samples were measured in triplicates.

### Invasion assay

Transwells (8 μm pore size, Corning) were coated with Matrigel^®^ matrix (30 µg/insert; Corning) for 2 h in 37^°^ C. RASFs (3.5 × 10^5^/6-well) were transfected with miR mimics, scrambled or siRNAs and mechanically strained as described above. Cells (5 × 10^4^) were seeded into the upper chamber of the transwell in serum-free conditions and DMEM containing 10% FCS (200 µl) was used as a chemo-attractant in the bottom chamber. After 24 h incubation, non-migrating cells were removed from the upper side of the porous membrane with a Q-tip. Invading cells were washed with PBS, fixed with 4% PFA for 5 min and stained with DAPI (2 µg/ml) for 10 min at room temperature. The whole 96-well was photographed with the EVOS FL microscope and all DAPI-stained nuclei counted using ImageJ.

## Electronic supplementary material

Below is the link to the electronic supplementary material.

Supplementary Material 1.

Supplementary Material 2.

## Data Availability

The datasets used and/or analysed during the current study available from the corresponding author on reasonable request.
