# Supplementary material for: Mechanical forces trigger invasive behavior in synovial fibroblasts through N-cadherin/ADAM15 -dependent modulation of LncRNA H19
Source: Sci Rep. 2025 Mar 21;15:9814. doi: 10.1038/s41598-025-94012-2 (PMC11928650; doi:10.1038/s41598-025-94012-2)
Supplement: Supplementary file 1 — Supplementary Material 1. [file 41598_2025_94012_MOESM1_ESM.pdf]

Supplementary Fig. 1

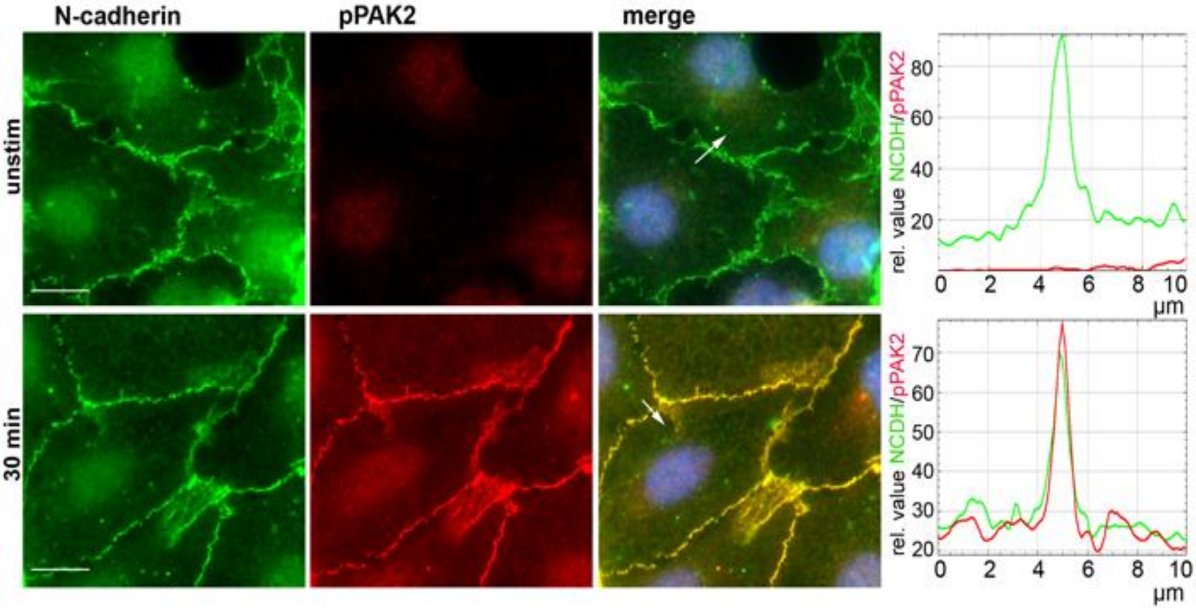

Suppl Fig. 1. Mechano-induced recruitment of pPAK2 to the cell membrane. RASFs grown on Bioflex plates and either unstimulated or strained for 30 min were fluorescently stained for NCDH and pPAK2. Adjacent to images: quantification of the pixel density of NCDH /pPAK2 along the white arrow. Cell nuclei were counterstained with DAPI. Objective 40x, scale bar = 10 µm.

Supplementary Fig. 2

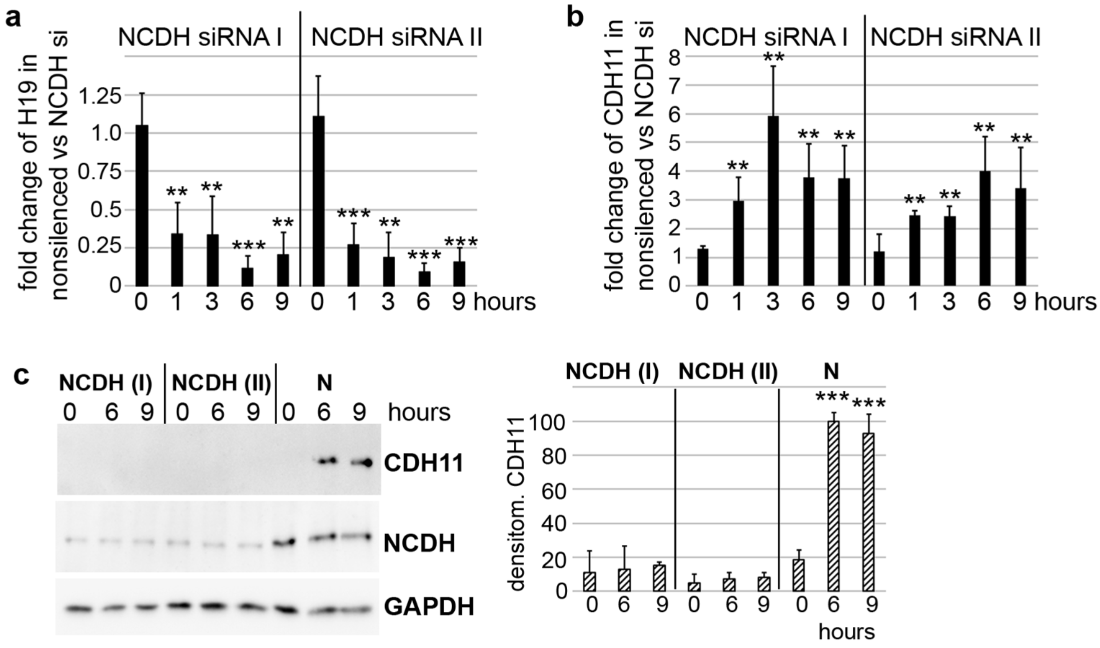

Supplementary Fig 2. LncRNA H19 and Cadherin-11, but not Cadherin-2 (NCDH) is a target of mechano-repressed NCDH. a-c RASFs silenced with NCDH siRNA (I), siRNA (II) or nonsilenced (N) were mechanically strained and **a** RT-qPCR for H19, and **b** RT-qPCR for

CDH11, fold changes from 5 different RASFs (mean ±SD) **c** immunoblots for CDH11 and NCDH, right panel, densitometry from 4 different RASFs. GAPDH served as loading control. **p < 0.005, ***p < 0.0005, Student's t-test.

Supplementary Fig. 3

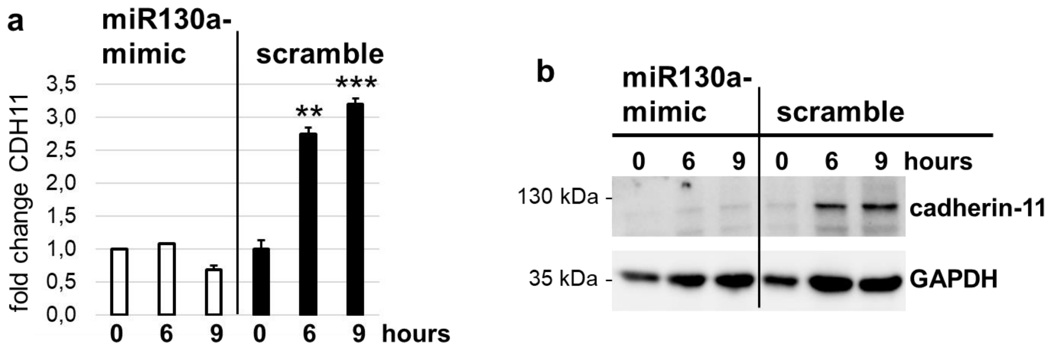

Supplementary Fig 3. Influence of miR-130a-5p on cadherin-11 expression. A) RT-qPCR for CDH11 from RASFs (mean ±SD from 3 donors) prior transfected with miR-130a-5p and scramble mimics and mechanically strained for 6 and 9 hours. **B)** immunoblots for CDH11 from RASFs prior transfected with miR-130a-5p and scramble mimics. Shown is a representative result from 3 independent experiments. GAPDH served as loading control. **p < 0.005, ***p < 0.0005, Student's t-test.

Supplementary Fig. 4

a)

https://www.mirbase.org

Homo sapiens **hsa-mir-130a precursor miRNA (89 nt)**

hsa-miR-130a-5p **hsa-miR-130a-3p**

. . . . AGCUCUU**UUCACAU**UGUGCUAC . . . .
. . . . AG**CAGUGCAA**UGUUAAAAGGGCA

Seed sequences are highlighted in bold.

Homo sapiens hsa-miR-130a-3p mature miRNA (MIMAT0000425)

miR-130a-3p seed: 3′ **AACGUGA** 5′

hsa-mir-130a-3p 5′ **CAGUGCAAUGUUAAAAGGGCAU 3′**

seed 5′ **CAGUGCAA** 3′

3′ **AACGUGAC** 5′

||||||||

gene level 5′ **TTGCACTG** 3′

Homo sapiens hsa-miR-130a-5p mature miRNA (MIMAT0004593)

5′ - GCUCUUU**UCACAU**UGUGCUACU – 3′

3′ CGTGAA**AAGTGTA**ACACGATGA – 5′

gene level 5′ **ATGTGAA** 3′

b)

https://www.ensembl.org/Homo_sapiens/Transcript/Exons?g=ENSG00000140937;r=16:64943753-65122063;t=ENST00000268603

>CDH11-201 utr3:protein_coding

CAATAACGATACAAATTTGGCCTTAAGAACTGTGTCTGGCGTTCTCAAGAATCTAGAAGATGTGTAAACAGGTATTTTTTTAAATCAAGGAAAG
GCTCATTTAAAACAGGCAAAGTTTTACAGAGAGGATACATTTAATAAAACTGCGAGGACATCAAAGTGGTAAATACTGTGAAATACCTTTTCTC
ACAAAAAGGCAAATATTGAAGTTGTTTATCAACTTCGCTAGAAAAAAAAAACACTTGGCATACAAAATATTTAAGTGAAGGAGAAGTCTAACGC
TGAACTGACAATGAAGGGAAATTGTTTATGTGTTATGAACATCCAAGTCTTTCTTCTTTTTTAAGTTGTCAAAGAAGCTTCCACAAAATTAGAA
AGGACAACAGTTCTGAGCTGTAATTTCGCCTTAAACTCTGGACACTCTATATGTAGTGCATTTTTAAACTTGAAATATATAATATTCAGCCAGC
TTAAACCCATACAATGTATGTACAATACAATGTACAATTATGTCTCTTGAGCATCAATCTTGTTACTGCTGATTCTTGTAAATCTTTTTGCTTC
TACTTTCATCTTAAACTAATACGTGCCAGATATAACTGTCTTGTTTCAGTGAGAGACGCCCTATTTCTATGTCATTTTTAATGTATCTATTTGT
ACAATTTTAAAGTTCTTATTTTAGTATACGTATAAATATCAGTATTCTGACATGTAAGAAAATGTTACGGCATCACACTTATATTTTATGAACA
TTGTACTGTTGCTTTAATATGAGCTTCAATATAAGAAGCAATCTTTGAAATAAAAAAAGATTTTTTTTTAATTCTGGGTTTGATTCTTAACATT
GAAACAAACGTTAAGTATTTCTAATGATCCATTTATATTTCTAATTTAATTGTGATCTTTTAATAACCCTATTTATGATCTGTTGTTGTCTGTC
TGCTGCTTTTATTGTTTATTTAAAATCAAATATGTTTTACAAATGTTTTTTCAGACAAGATTCTGTAACATCATGTAAAGCTTTTTTGTACATT
CTTGGTGTTAACCTCCTGGCTTCTCTTCACACACATCTTCTAAAAAAGAAGG***ATGTGAA***AGAACTAGGTCAGTCTATGACTTTGCAATAT
GTGTTATATAGTATGCATTTATCTTGTATATCAGTAATTTGATGGTTATGAGAGATGAATCCATGAGGGAATGGAGCTATCAGAACTCTAATGT
TCCAGGTATACATTCTATGCCCCACACTGAGCACTGGGGAACTGGGGGACTAGAGTCAAAAATATAAATTTGCCCAGACTCTAATGTTATTCTA
TTTTTTCTTCTGTTGAACTTACCAGGCTATTGTAAGACTCTTGATAGTTGAAACTGCTTATTTTTCCTCCTGTAATTTTAACTAATTGTAAAAT
GATGTGGCATTTTATGTTTTAATGAGAATGGGCGATTCATTTAAAAAAGCTTTGTTTAGAATATGCTTGGGGCCGTAAGCTCAGAATGAGGGCA
GGGACCATTTTGGATTCTGAGAGTCGATGCCATTTGGTCCAGGAGTGTGTCTACAGTCCCCTGCATTCCAGCTAGTTTCTTGGGGATTGAAACT
T***ATGTGAA***GGGCATTTCACCTGTTCAGTTGGGCCAAAGGTCAAAACGTAGCAATACTTGGGGAAAGACCACATAAAGTCACACTGCAAGT
GCTTTCCCTCTTTCCCCCTACACACAGGGCACGTGCTTTTTCTTGGATTGCAGACAATTTTTACAGTTTTTTTCTGACTTTATTGTGAAAGTTT
GTTTCAAGCATTTCTTGATATCATGTTATGTACTATTTTTATGATTTAGTCAACATGCATACAAAGAAATGTTTTTTATGAAGTGCTCACTTCC
ATTTTACTTTGCATTGAAATCAAATTGGGCTGAACACTTCAATGGAATACATTTTGTGGACAATGTCACTTTAGAATCTTTCATCTCAGTGAAG
GATTACACATTCTCAATACTTCCATAATTGCAGGTTGTGTTCATTTTTTTATATAGTTTTTGTAATCCAAAGAATATTTTGCTAGAT**TTGC**
ACAGATCTCCAATTGAATTTGCAATGAAGAAATAACTCAAAAGGAATATGAATAGCATTTAAATAAGTATACAGCTGTAAGTAACCCTGTCA
CCATGGATGATCCTTTTCTCTAGGAATGTATTTGGATTAGAGATGACAACTACATTTTCGCATTTTTATGTTGAAGTCTTTTTTAAAAAGGCTG
TTTACTTTTCAGTAGTTAAGAATACTTGTTTTTCTTTTTCTTTTTTTTTTTTTTTTTTACCTTTTATTTTTTCGTTAAGCCTCTATTGTTTGTA
GAACACTCTTAGAAACTTGGAAATAAAATGTCTTTCCCAACTAGTGGAGTCCTTTTTCATTTGGAGCACATTGCCTTAAAAGAAGTCTTAATTT
AAACGGTCCTTCCTTATTCTAAAGTAATCACTGTTTTATACCATCTATGCAGCTAAAAGAAGGAACATGCTTCTGTTCTTTTCCTCAAGTAATG
G||TTATTGTTTCTAGTCATCATTCATTCATTGATTCATTCATTAATTCATCAAAATCTTATTTTATAAACCCTGTTCCACTTACTGGAGGATT
CAGAATGAATCTTACTACCTTTTCTGACATCTTTTGATAATTCAGCCCTGTACCAAAGTATCCACCTTGTTGTCTTATAATCACCTATTTACCT
ATTTGCCCTCCTAGAAAATGCAAGAAGATATTTTCTCTCCTTCCAAATTGAAGGAAGAACATAAAAGATATAACAGGAAGGAGATGGTGAGATA
TAGAGTGTGAGCGGAAATTAGGCCAGCTGTGGCAATTCTGGACAGATCTTGGGTTTAGCTAAGTTATTTCTTTTAGGCCTGGGTTTCTGGGGGT
GACAGGGAAGATAAAAGAGTAGTTTAT**TTGCAC**CTCTTGGAGAATTGCTTAAAAATATAGAGATCATGGCTCTGTATGTCAGGTGGAAC
CAGGTCAGGAGTATTTGAAACTGCTCCTGGGTCATTGTGACATATCCTTCACATCTTTTTGAGAAACTTTATAAGACAATGGGGGTGAATGGGG
GCTGGGCAGTTGGAGTCTCTGAGCAGAAGAGGGGCAAAATTTATTTGGCAGGCAGTGTGGAGGACAGATTAGGAGCATATAAACCCAGAGGTGT
GCCCCAGGAGGGCTTTTGCAAAGGTCAATATGAGATAGAATGAGGGCCTGAAATAATTCAGTAATTTGGAGATGGAGAAGAGGAAAGACTTCTC

TGCTC**TTGCACTG**CCATCAGCCTGGTCTGGGCCATGGTCATCTCTGACCCGGAAGACTGACCCCACCTCTTGGCTCACCCTCTGCCTC
CCAACCTCCTCTTCACAAAGAAGCCAGAGGGATACTTTTAACACACAACCCAGATCACATGACTTCGTAACTTAAACCTCTTCACTGGCTTCCC
AAAGACTTAAAATGAATTCTGATGCCTTTATTTTATTGCTTTACATGAACAGGGCCCTGCGAACCTCTCCAGTGTCATTCCACTCCATCCTCCT
TTCAGTGCACGATGCTCCAGCCACACTGGCCATCTTTCGGTTCCTGATACAAAAAAAAACACGTTCCTTTTCCATGGAAAGCAGGTCACCCTTG
TTATTTTGTATCGATGACAACTCTTTAAACTTATTTTGCTTTTTGGCTTTATGTATGTGTGTGGGTGGGTGGGACTGACTGCCCCACTAGAATG
TAAGCTCCATGAGGGCAGGGAATCTTGCTTTCTTGTTTACCATTGTATACTCAGTTCTTTACACAGTGCCTGAAACATAACAGGTACACAATAA
ATATCTATTGAATGAAAGCAAA

Supplementary Fig. 4. Alignment of miR-130a-3p and miR-130a-5p with 3' untranslated region (UTR) of cadherin-11. a Sequences for precursor and mature miR-130a from mirbase.org. **b** sequence of 3' UTR of cadherin-11 (www.ensembl.org). The seed sequences of miR-130a-3p (one 8-seed and two 6 seeds) are shown in **bold and underlined**. The alignment of the seed sequence of miR-130a-5p (***ATGTGAA; two7-seeds)*** is shown in italics and bold.
