# Supplementary material for: Mechanical forces trigger invasive behavior in synovial fibroblasts through N-cadherin/ADAM15 -dependent modulation of LncRNA H19
Source: Sci Rep. 2025 Mar 21;15:9814. doi: 10.1038/s41598-025-94012-2 (PMC11928650; doi:10.1038/s41598-025-94012-2)

Fig 2A

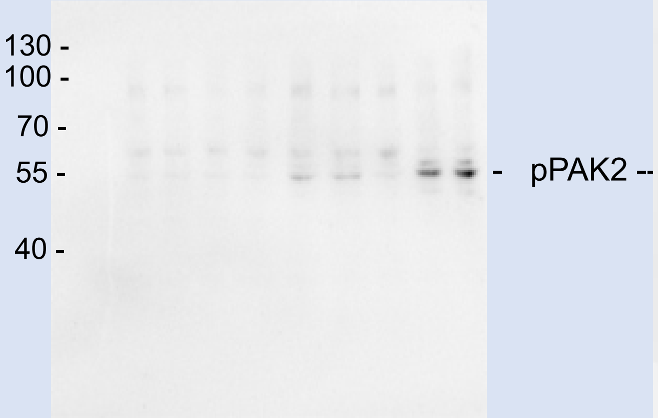

whole membrane

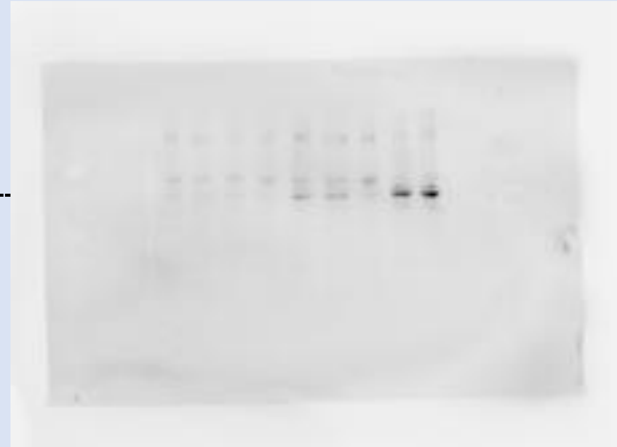

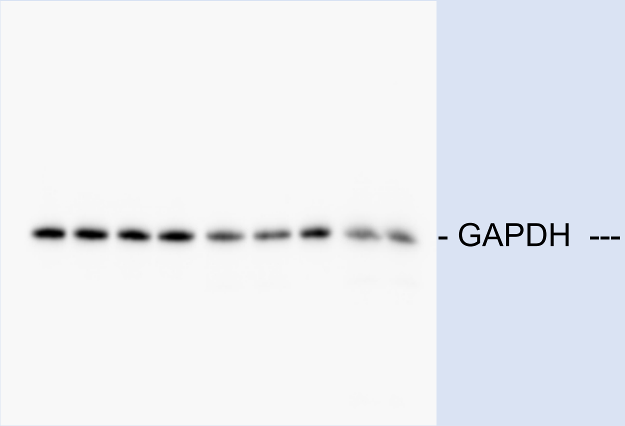

artificially merged overlay to see blot borders

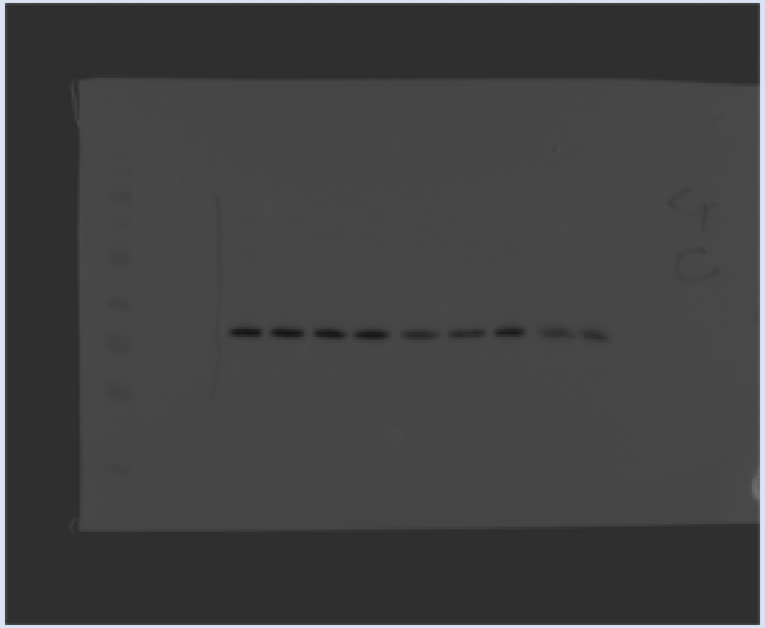

Fig 2B

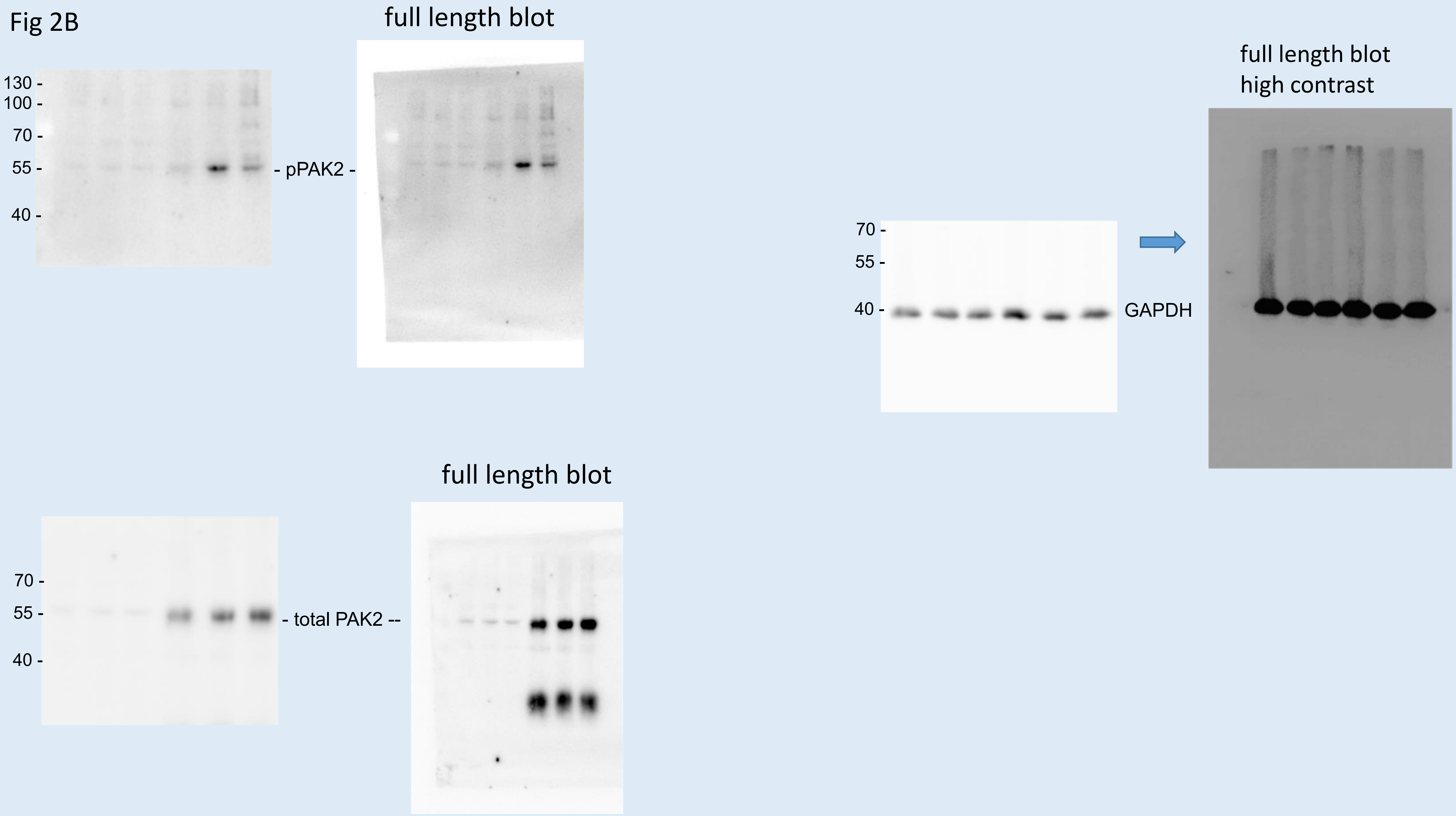
full length blot
130 -
100 -
70 -
55 -
40 -
- pPAK2 -
full length blot
70 -
55 -
40 -
- total PAK2 --
full length blot
high contrast
70 -
55 -
40 -
GAPDH

Fig 2C

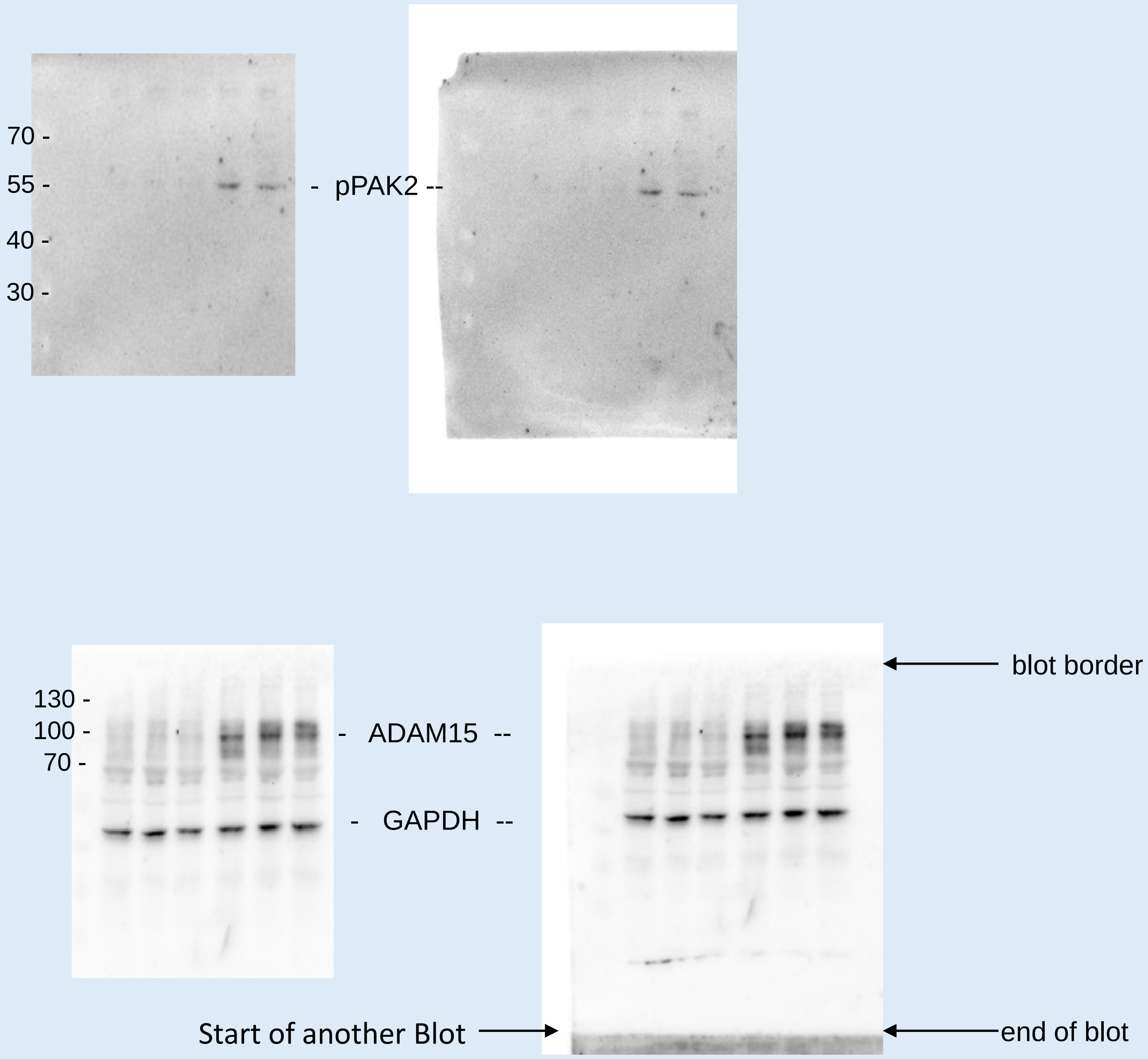
70 -
55 -
40 -
30 -
- pPAK2 --
130 -
100 -
70 -
- ADAM15 --
- GAPDH --
blot border
Start of another Blot
end of blot

Fig 2D

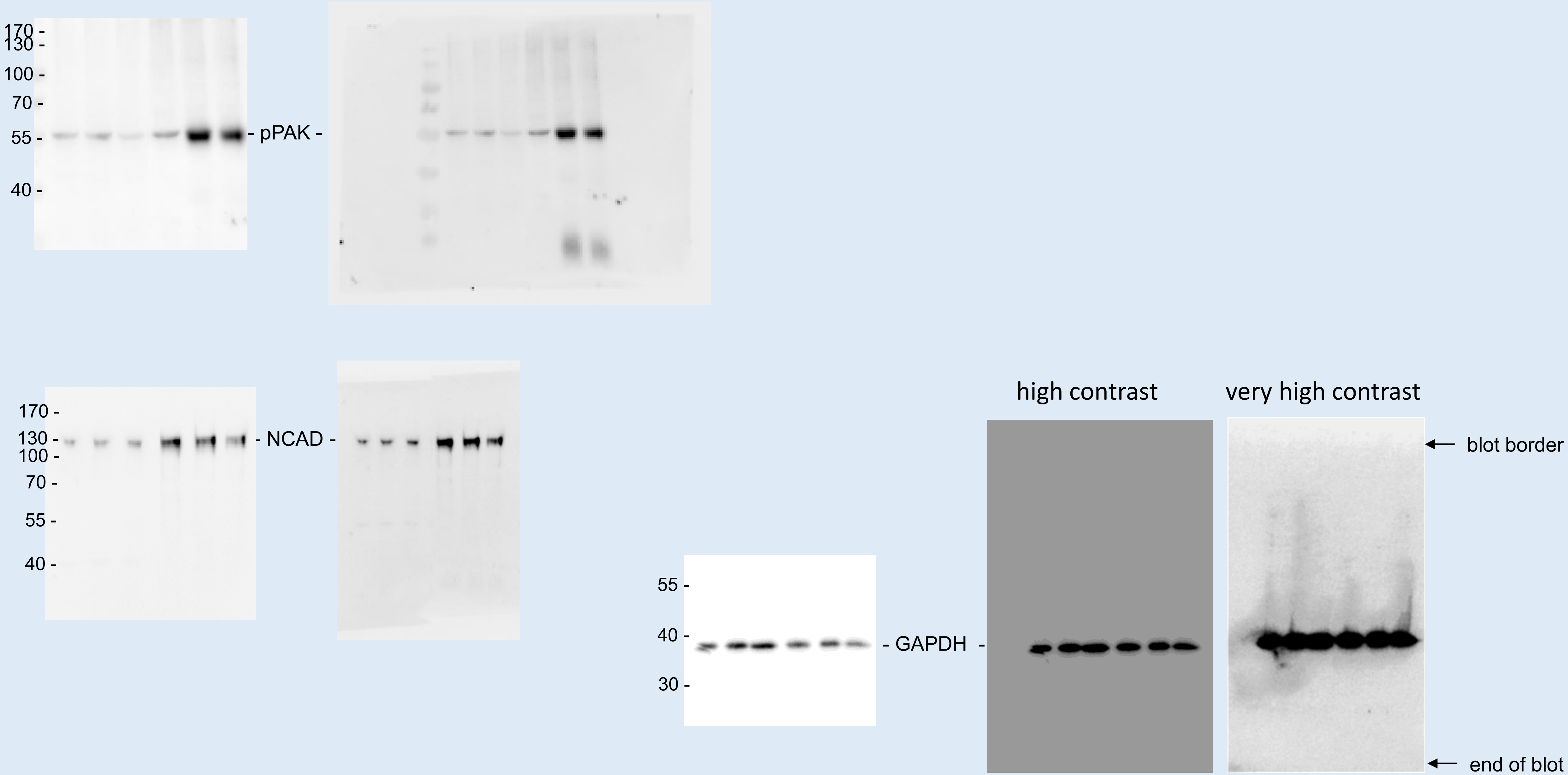
170 -
130 -
100 -
70 -
55 -
40 -
- pPAK -
170 -
130 -
100 -
70 -
55 -
40 -
- NCAD -
55 -
40 -
30 -
- GAPDH -
high contrast
very high contrast
blot border
end of blot

Fig 3A, upper panel

Blots with borders

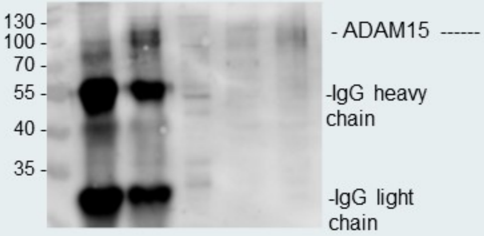

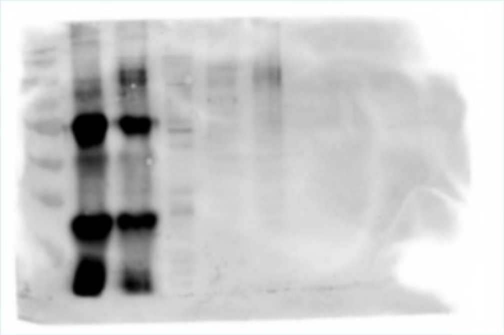

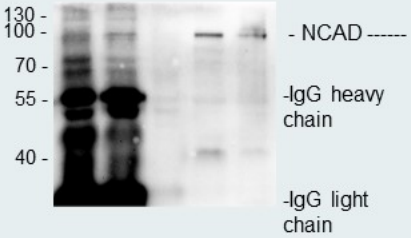

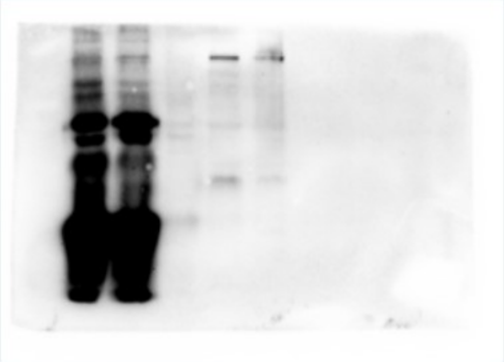

Fig 3A, lower panel

Blots with borders

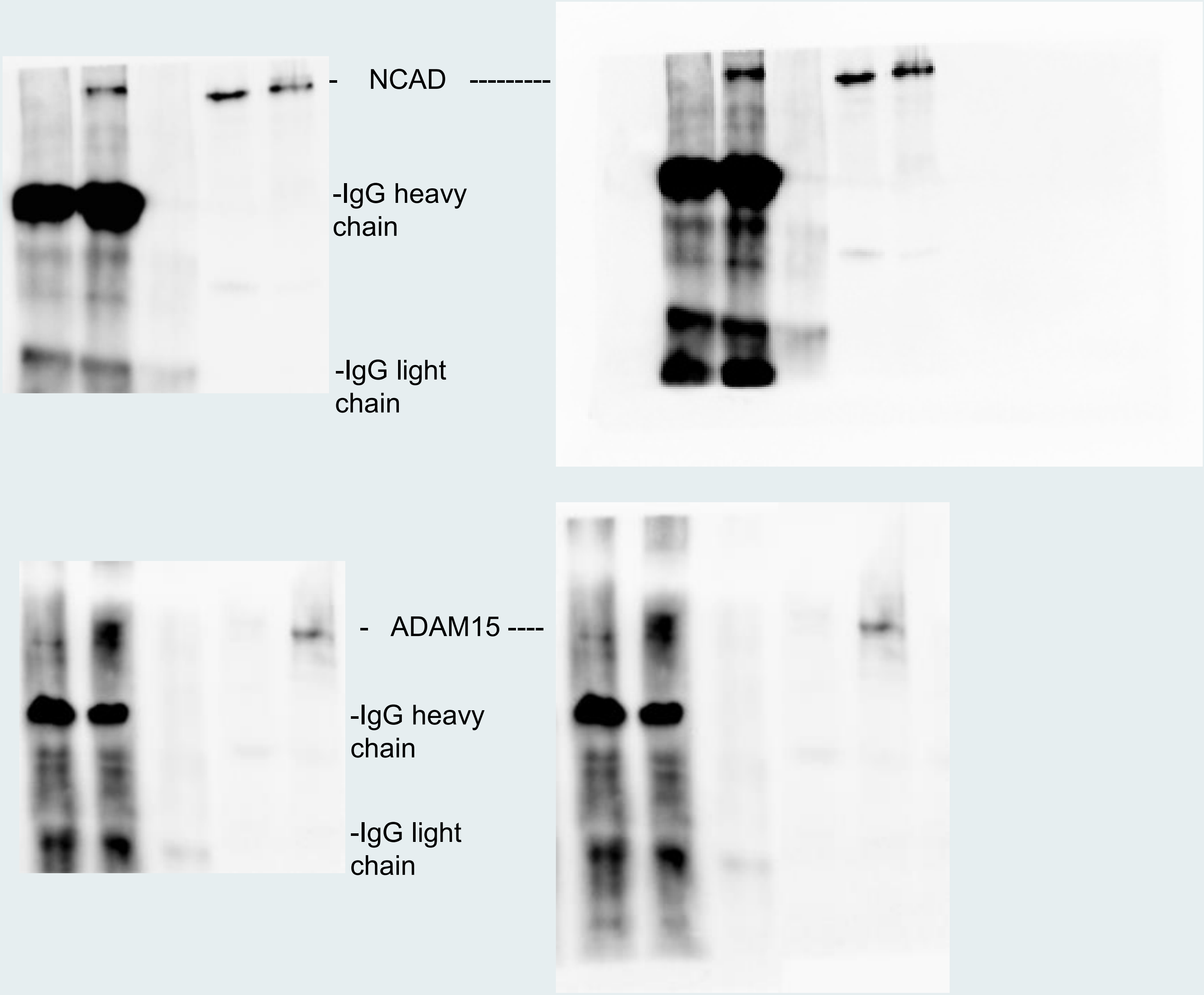

Fig 3B; upper panel

Blots with borders

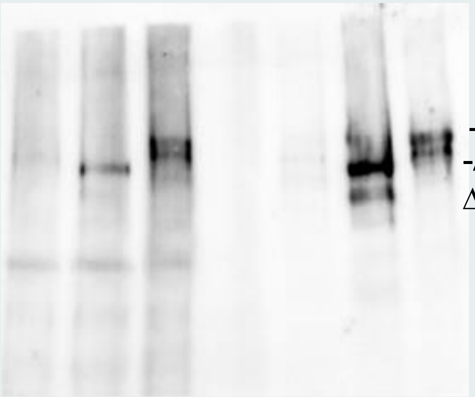

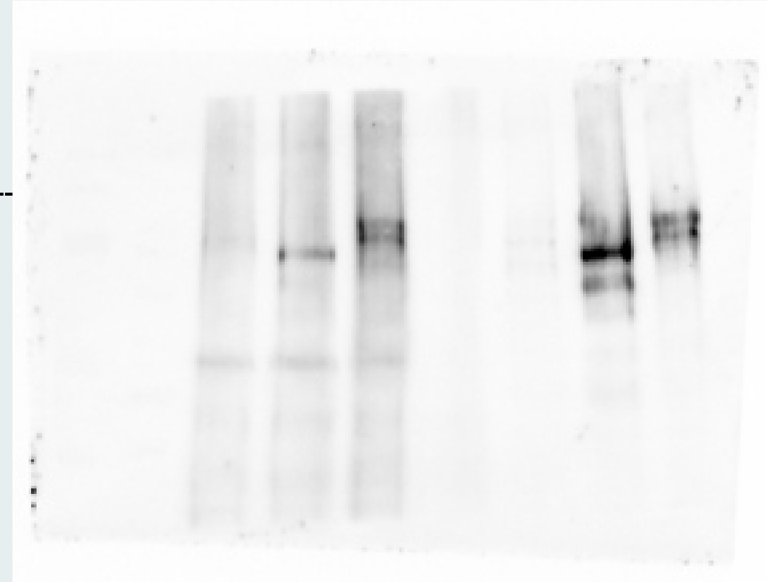

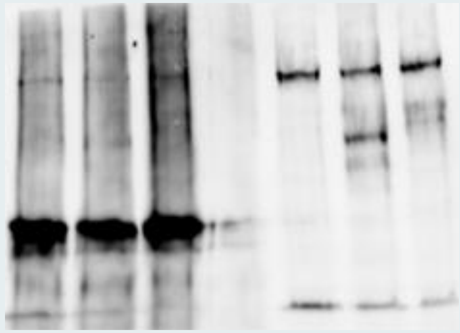

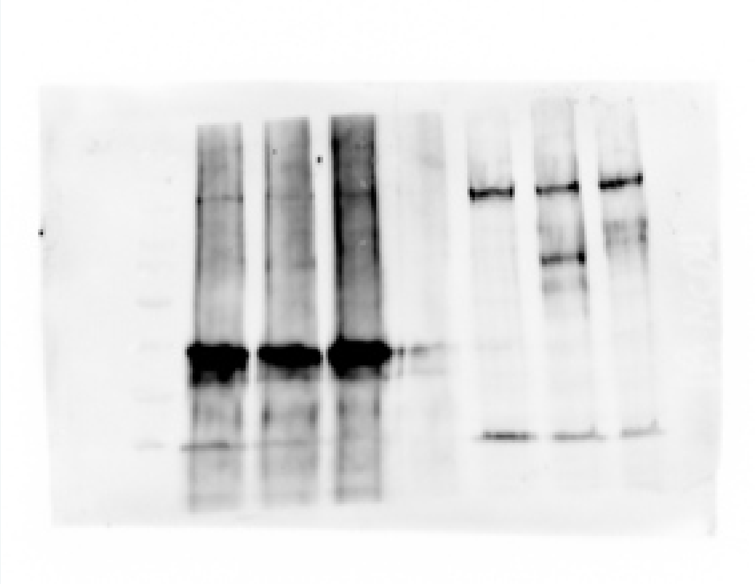

Fig 3B; lower panel

Blots with borders

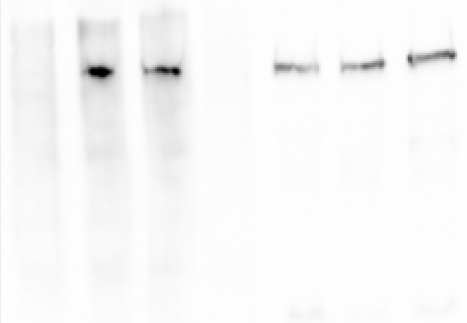

- NCAD --

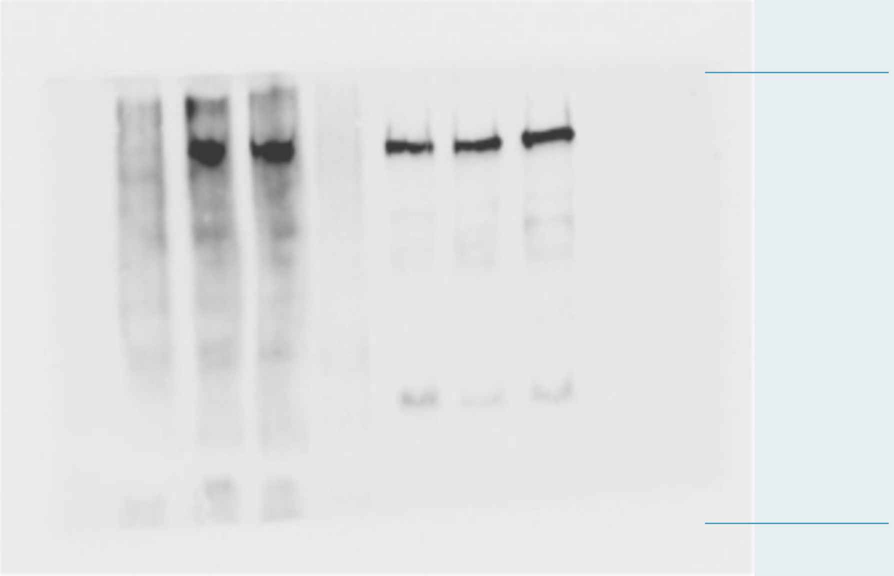

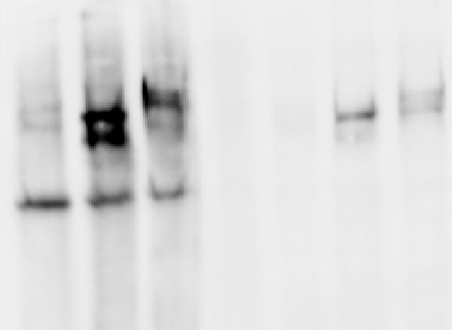

- ADAM15 --
-ADAM15 --
∆cyto

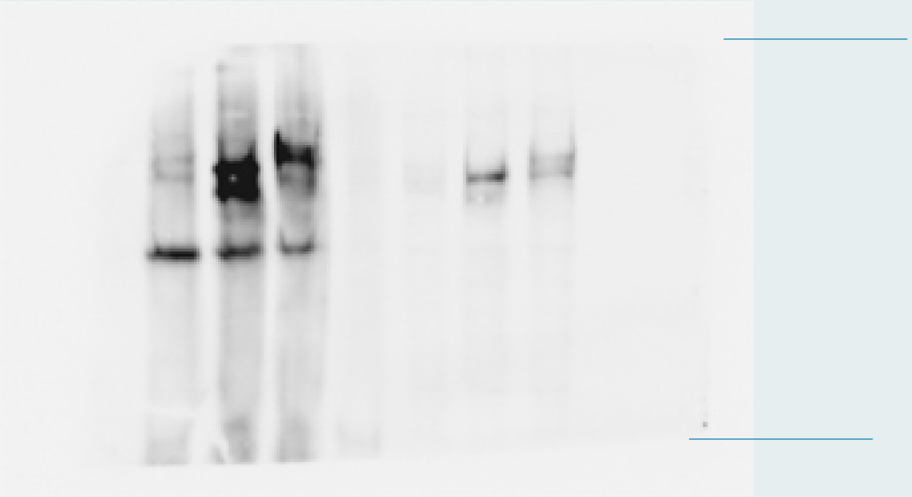

Fig. 4 D

whole full-length blots, start and end of blot marked by arrows

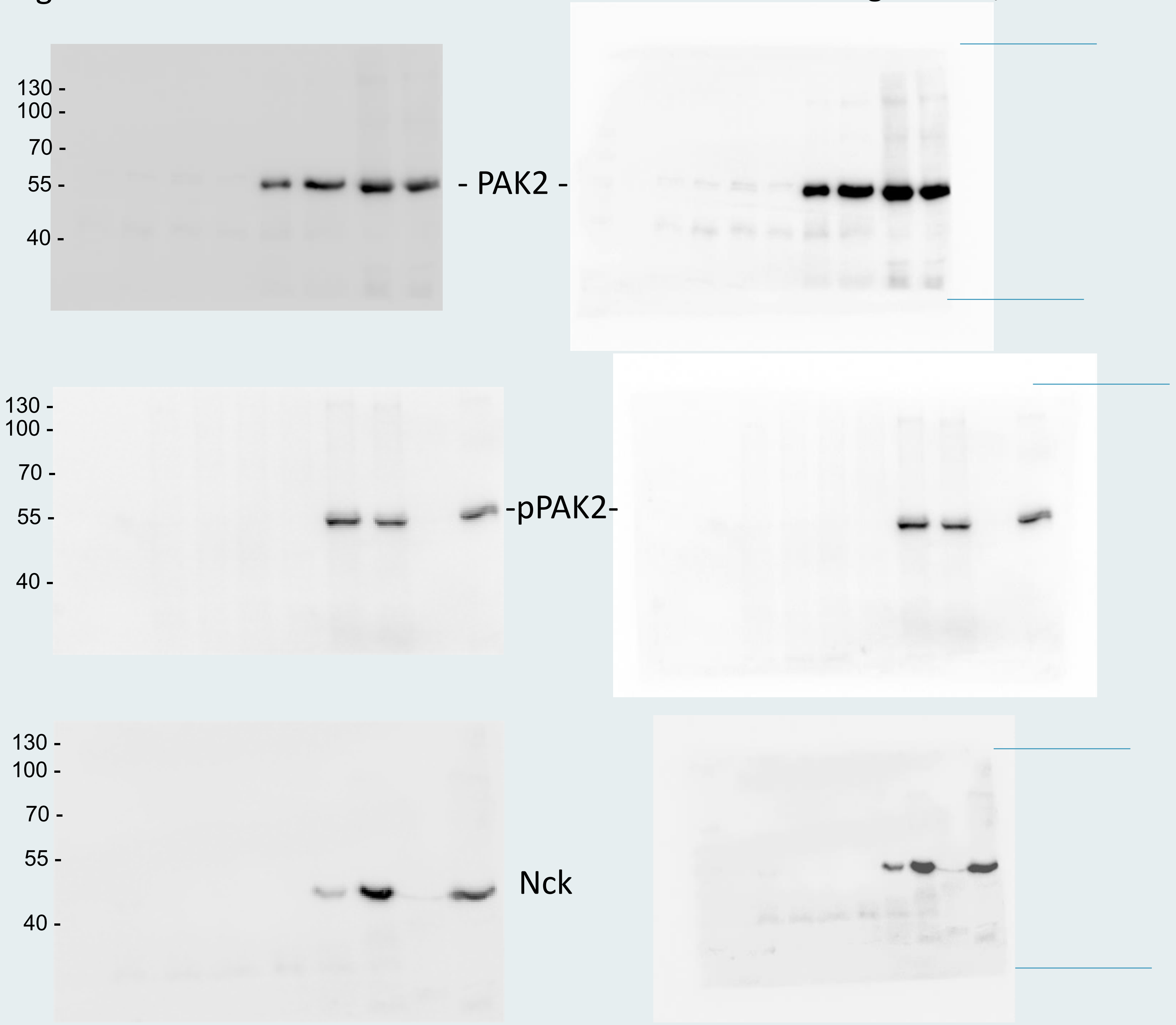

Fig. 4 D

Blots with borders

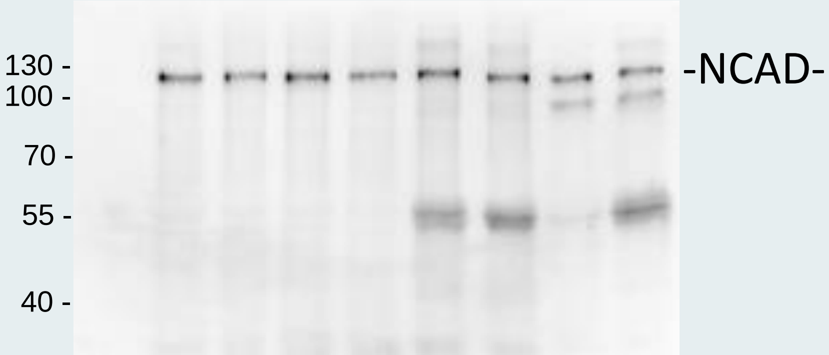

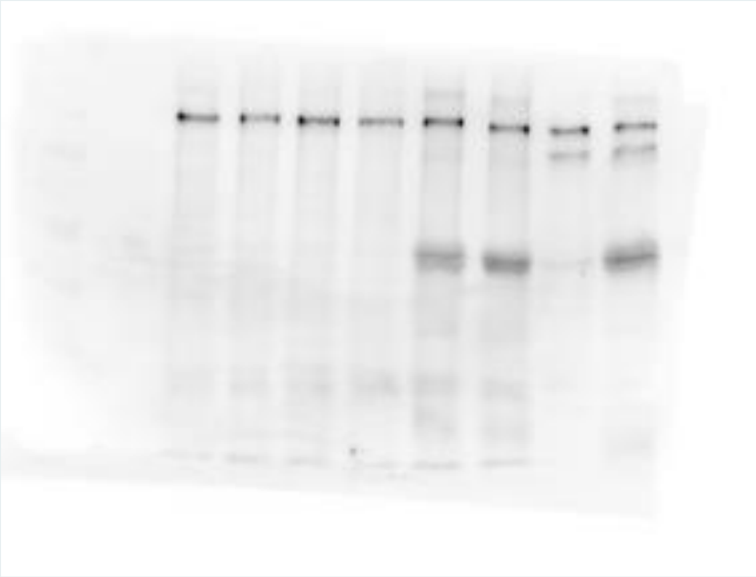

artificially merged image to show borders of Blots

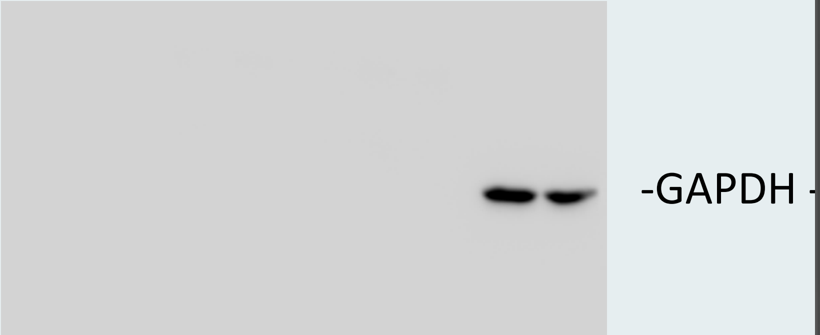

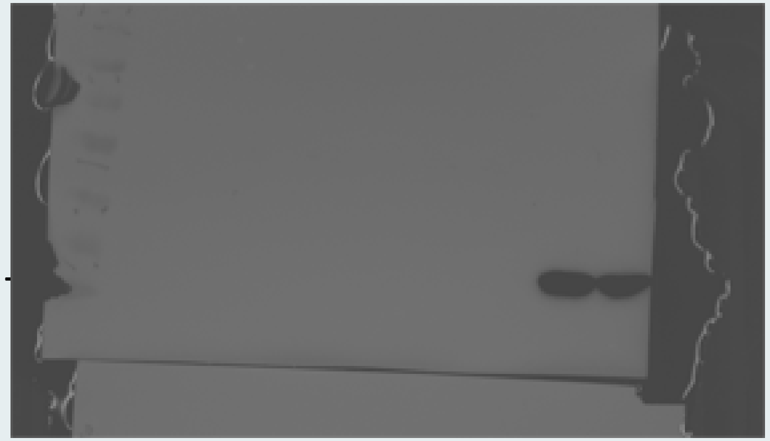

Fig. 5A

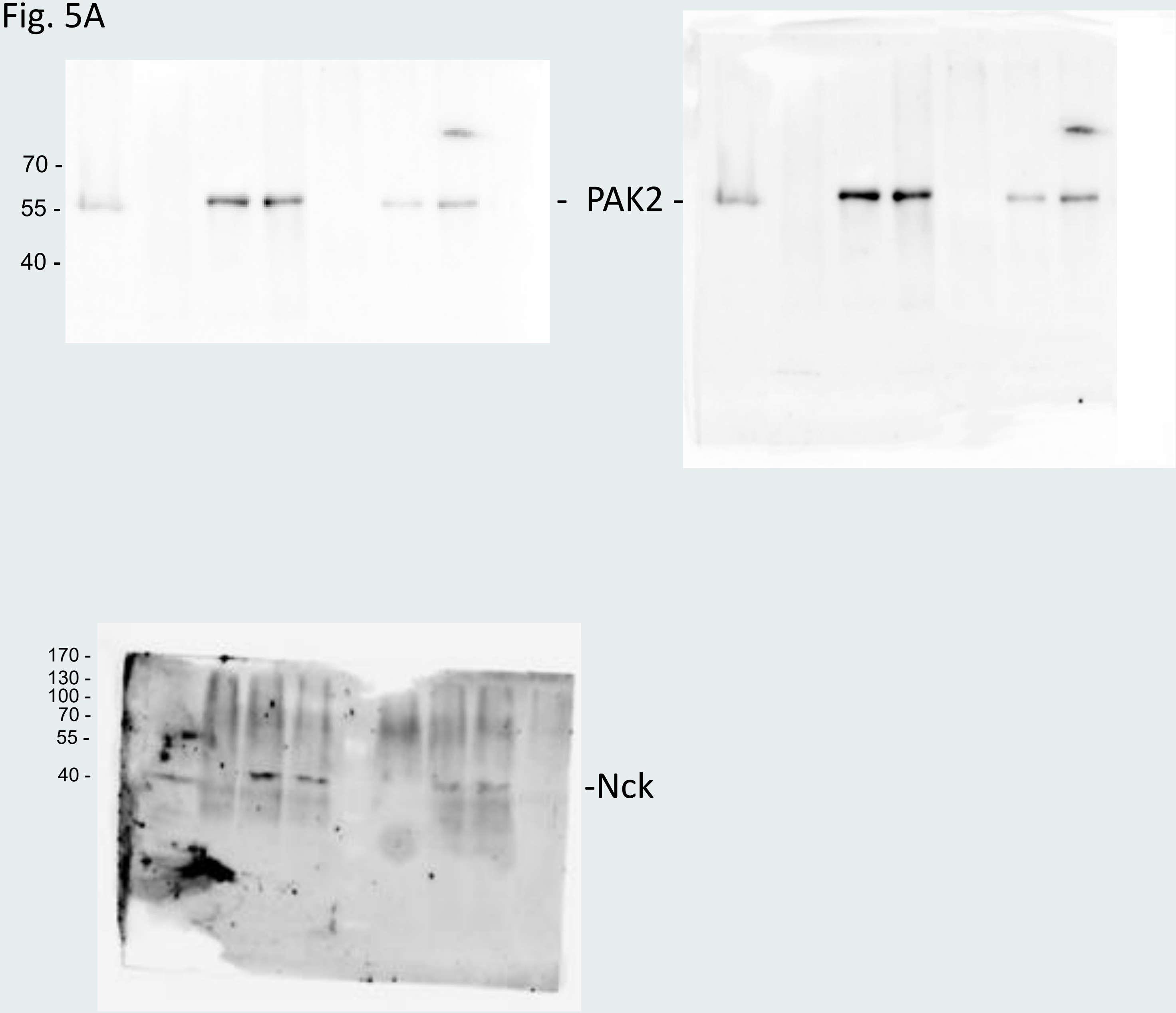

Fig. 5B

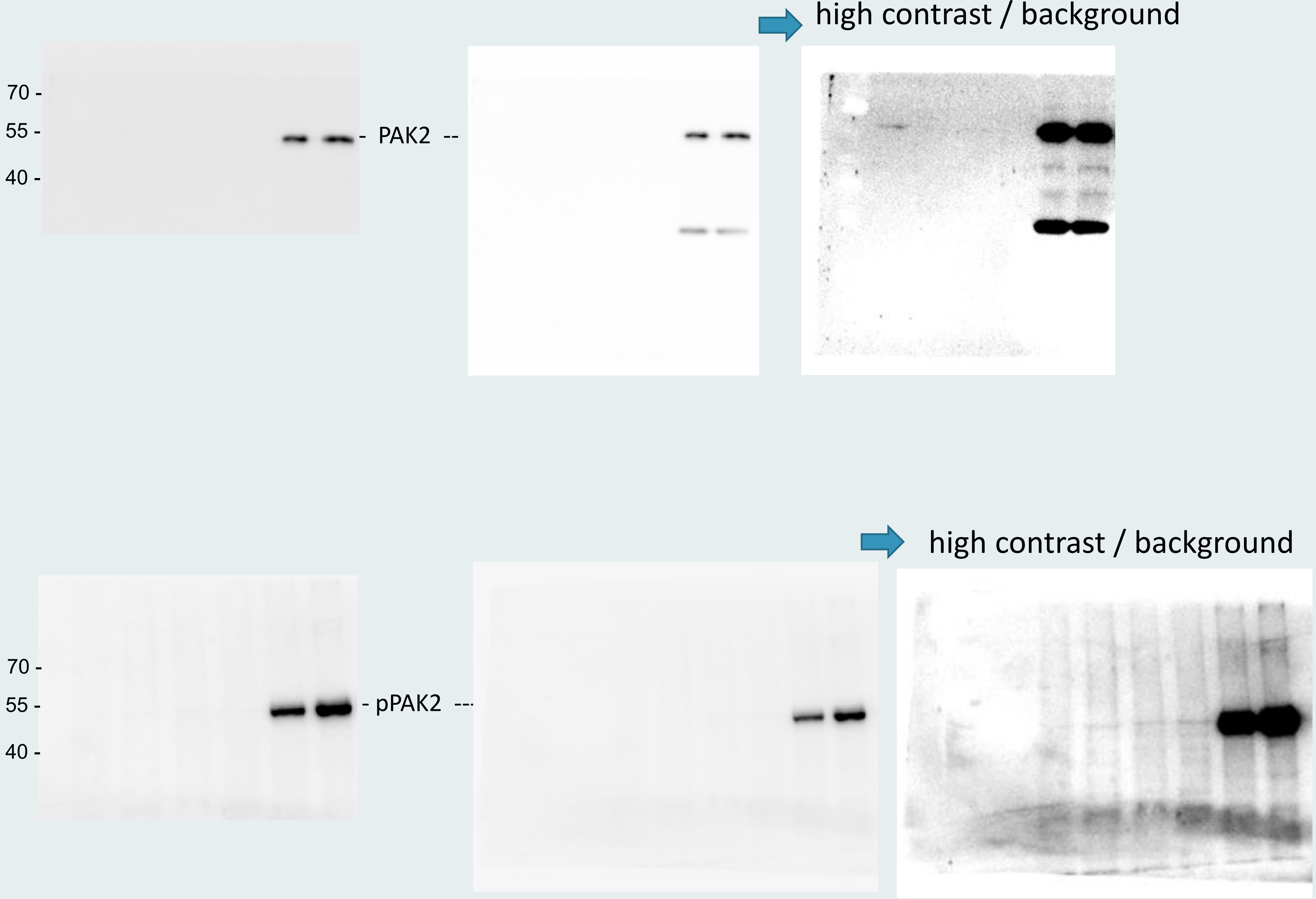
high contrast / background
70 -
55 -
40 -
- PAK2 --
high contrast / background
70 -
55 -
40 -
- pPAK2 ---

Fig. 5B

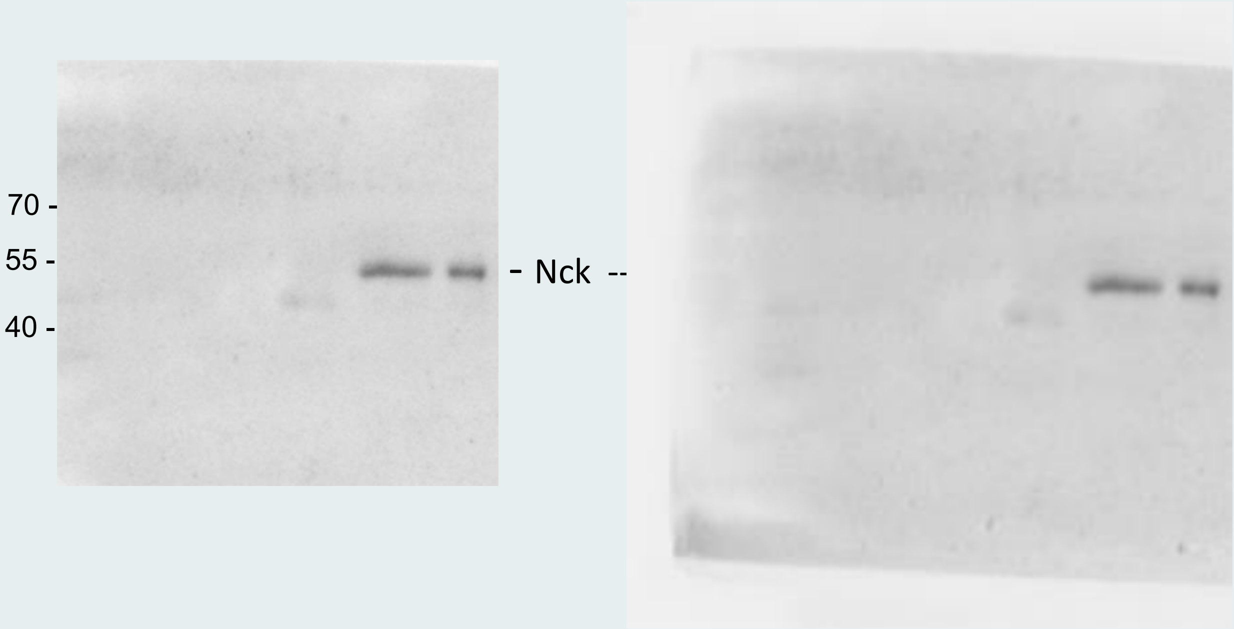

Fig. 5B

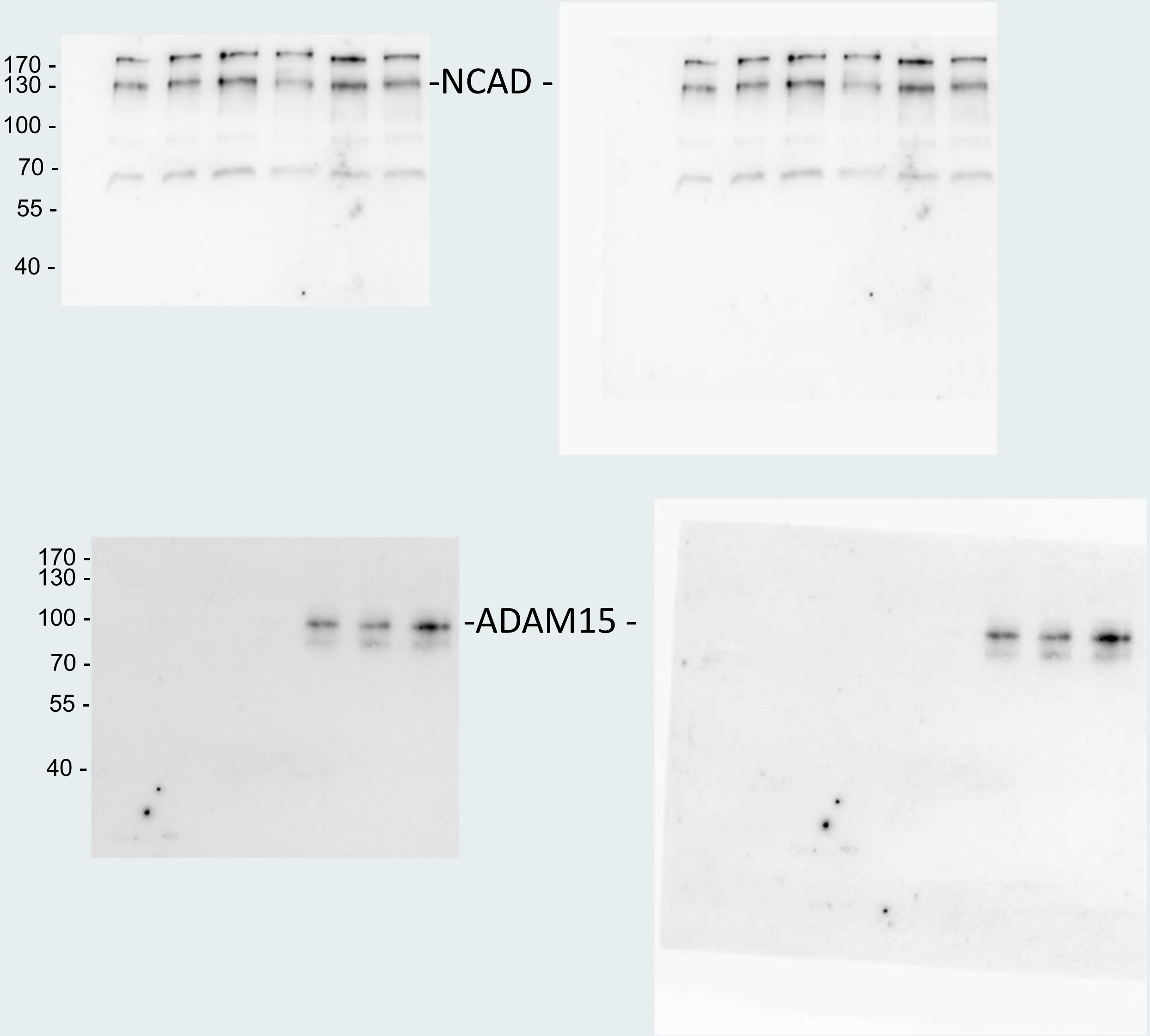

Fig. 5C

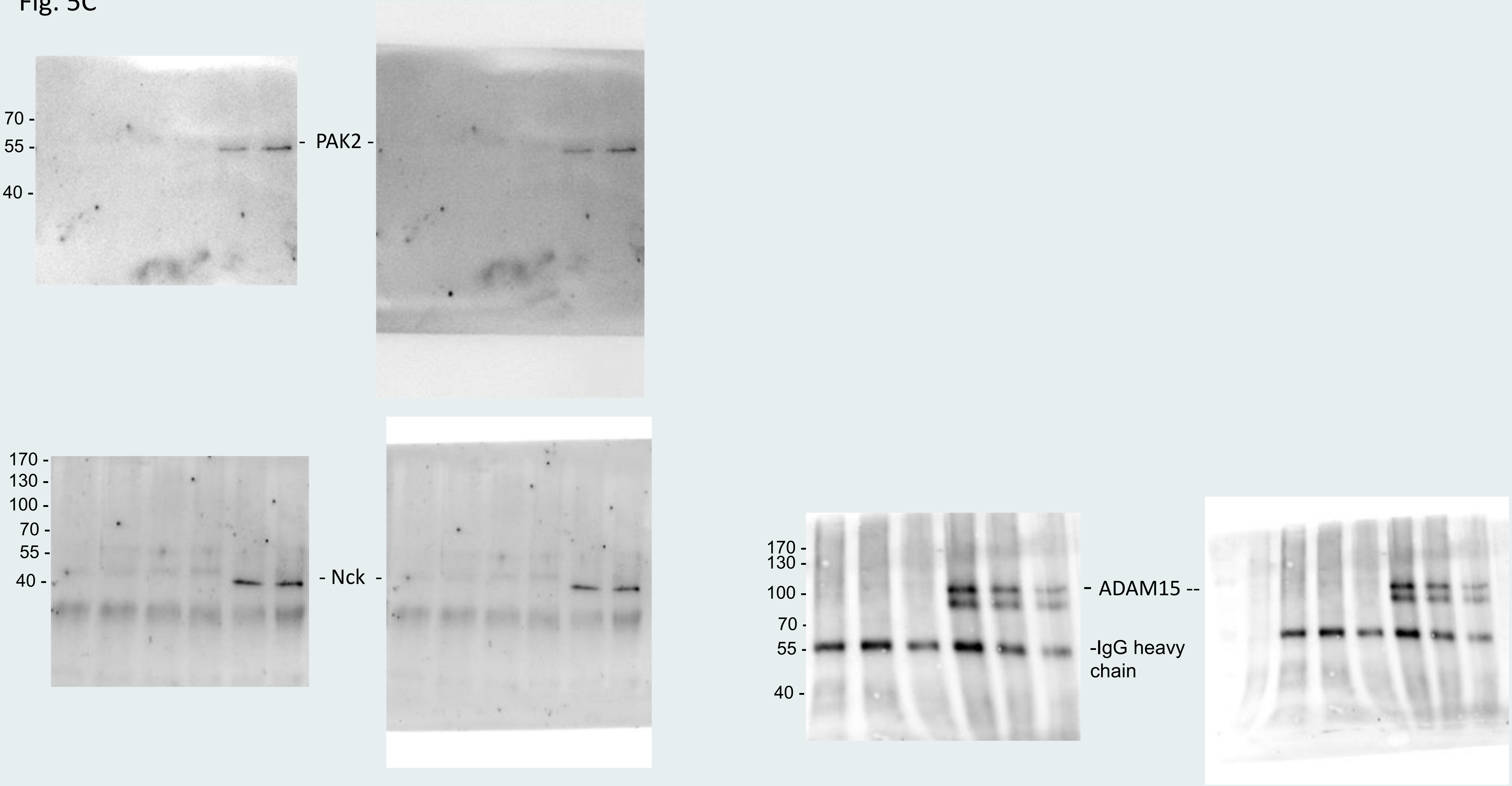
70 -
55 -
40 -
- PAK2 -
170 -
130 -
100 -
70 -
55 -
40 -
- Nck -
170 -
130 -
100 -
70 -
55 -
40 -
- ADAM15 --
-IgG heavy chain

Fig. 5D

full length blot → full length, high contrast/background to see border of blots

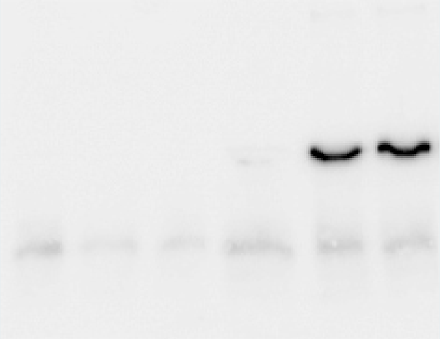

-PAK2 -

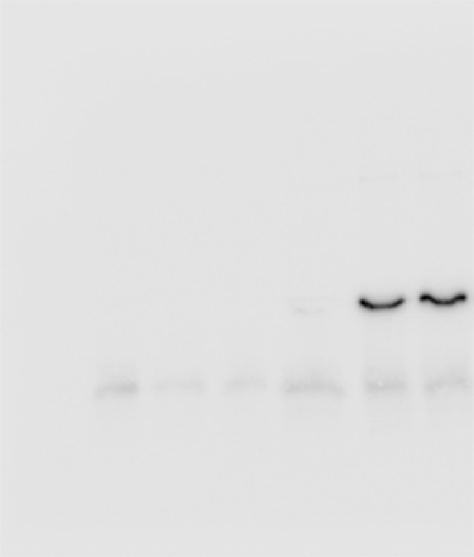

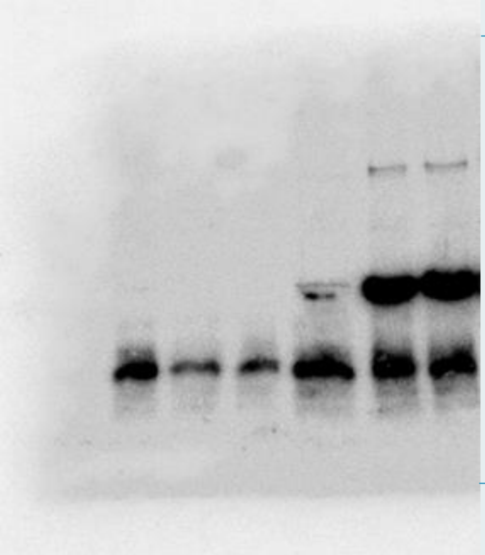

start of blot

-PAK2

end of blot

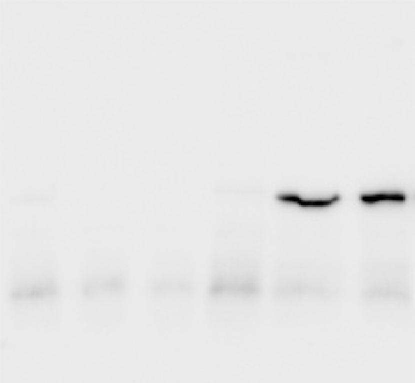

- Nck -

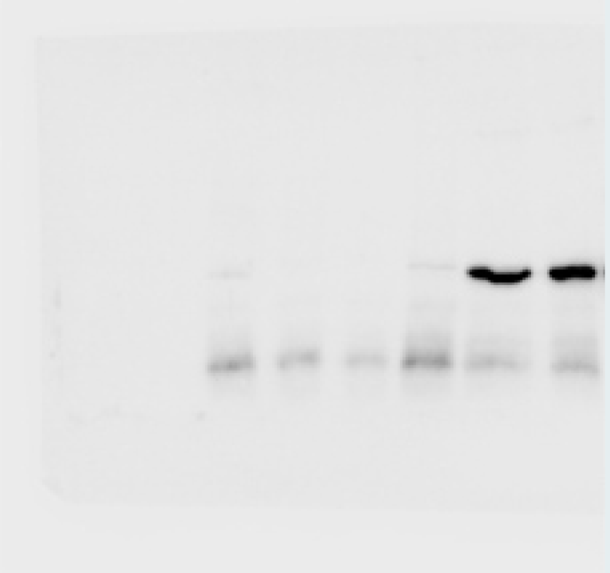

start of blot

end of blot

Fig. 5D

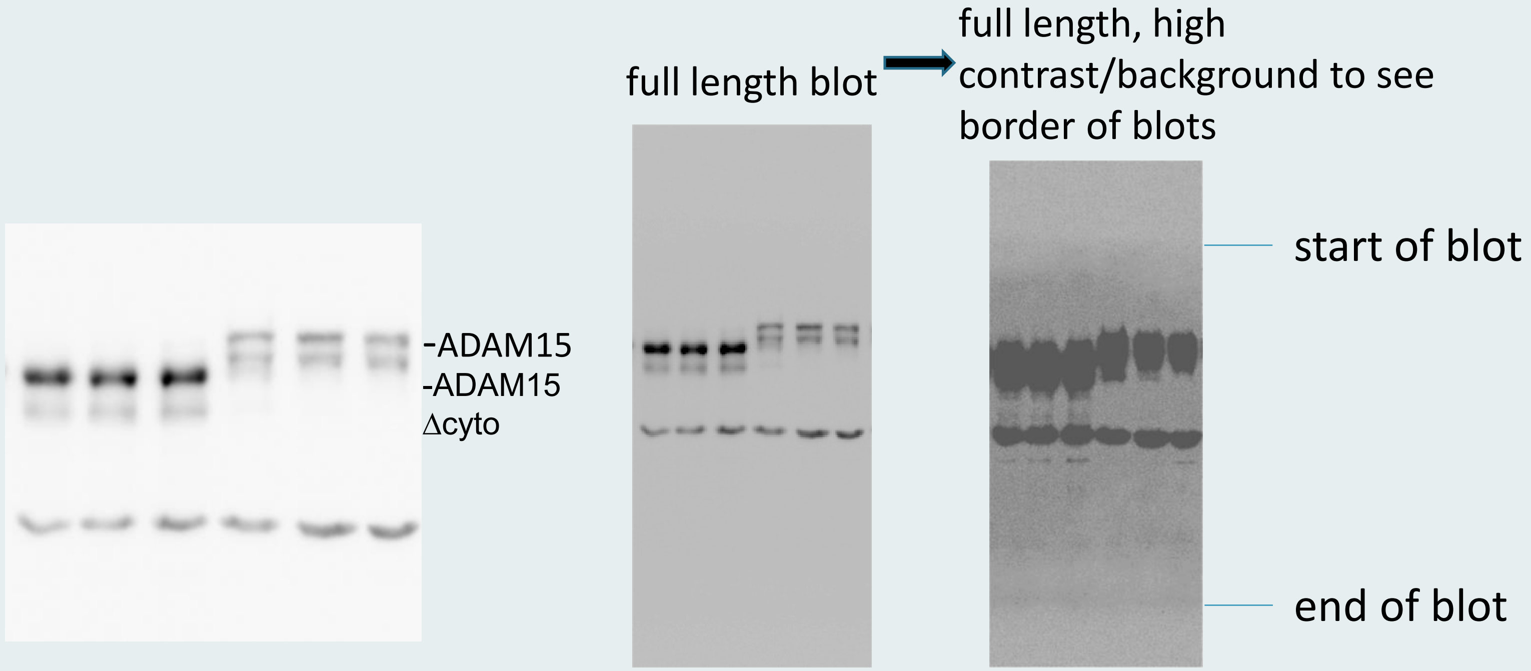

-ADAM15
-ADAM15
Δcyto
full length blot
full length, high contrast/background to see border of blots
start of blot
end of blot

Fig. 5 E

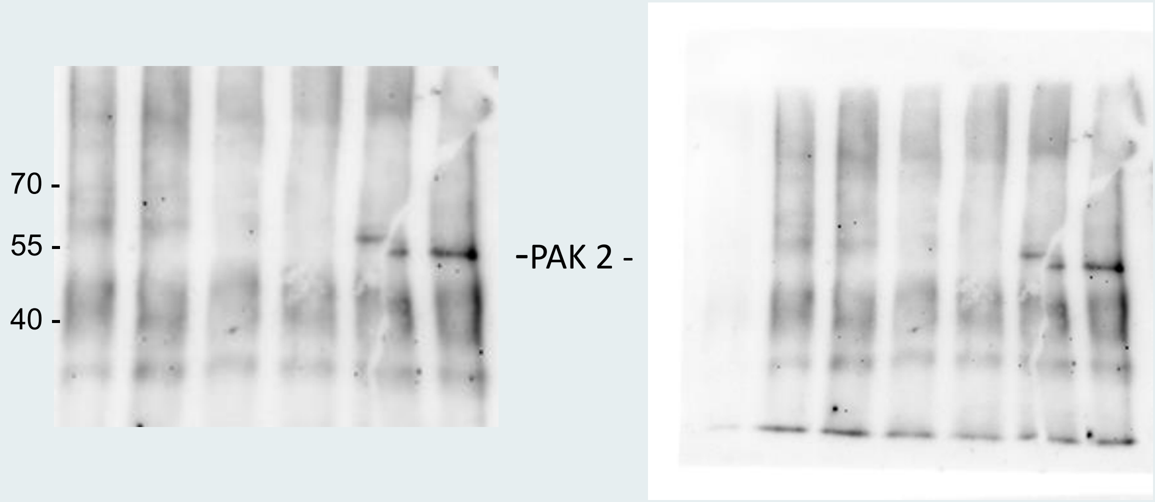

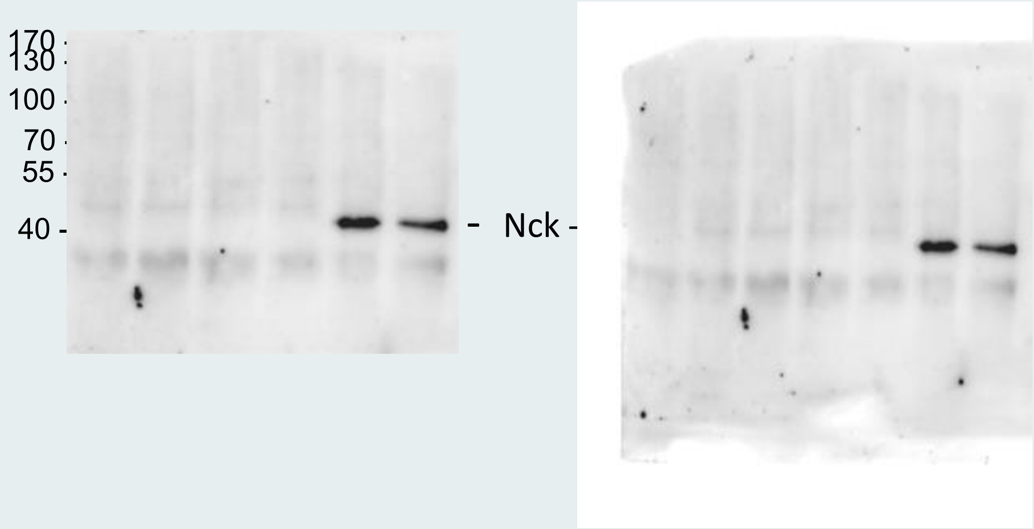

Fig. 5 E

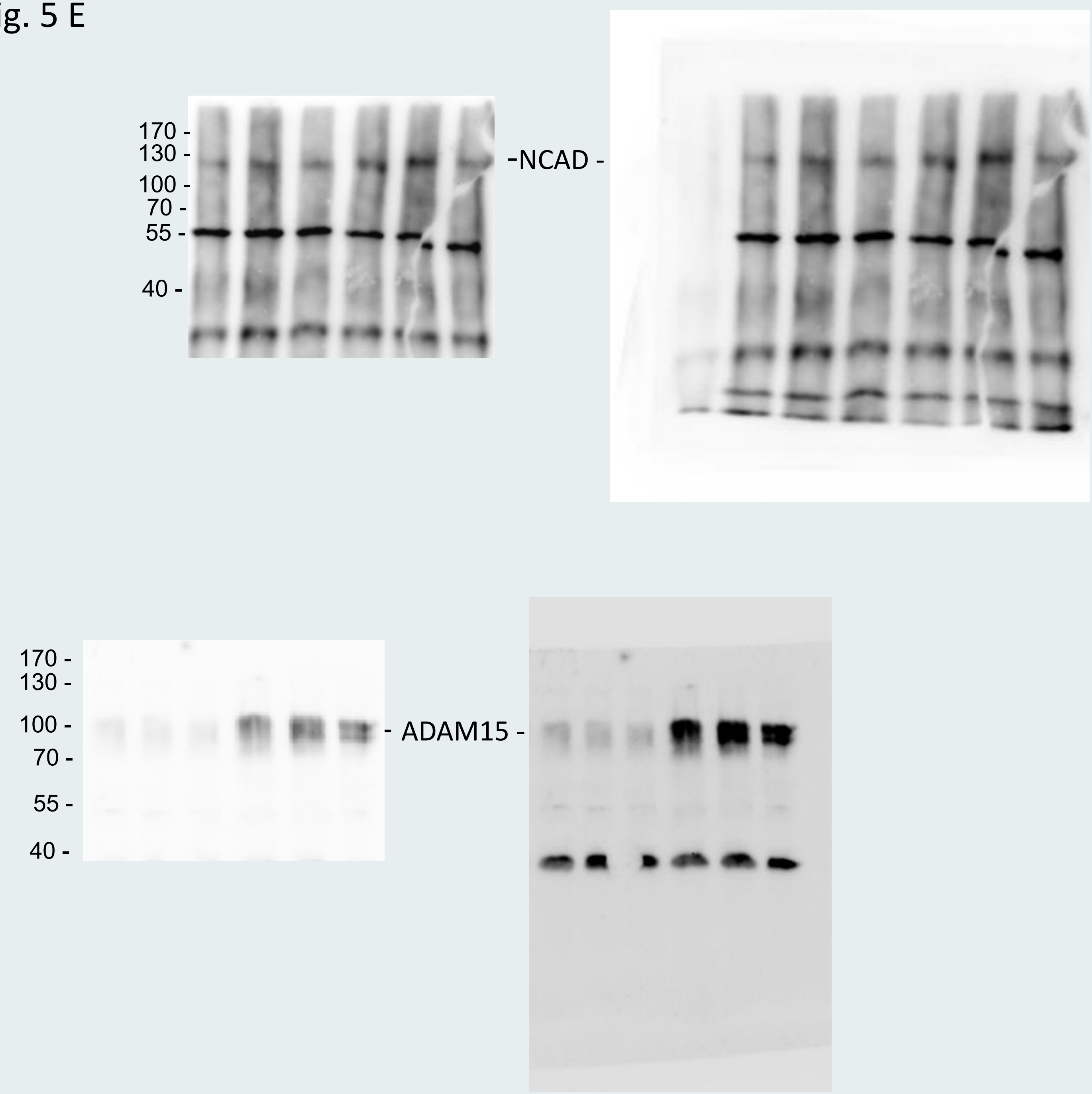

Fig. 5F

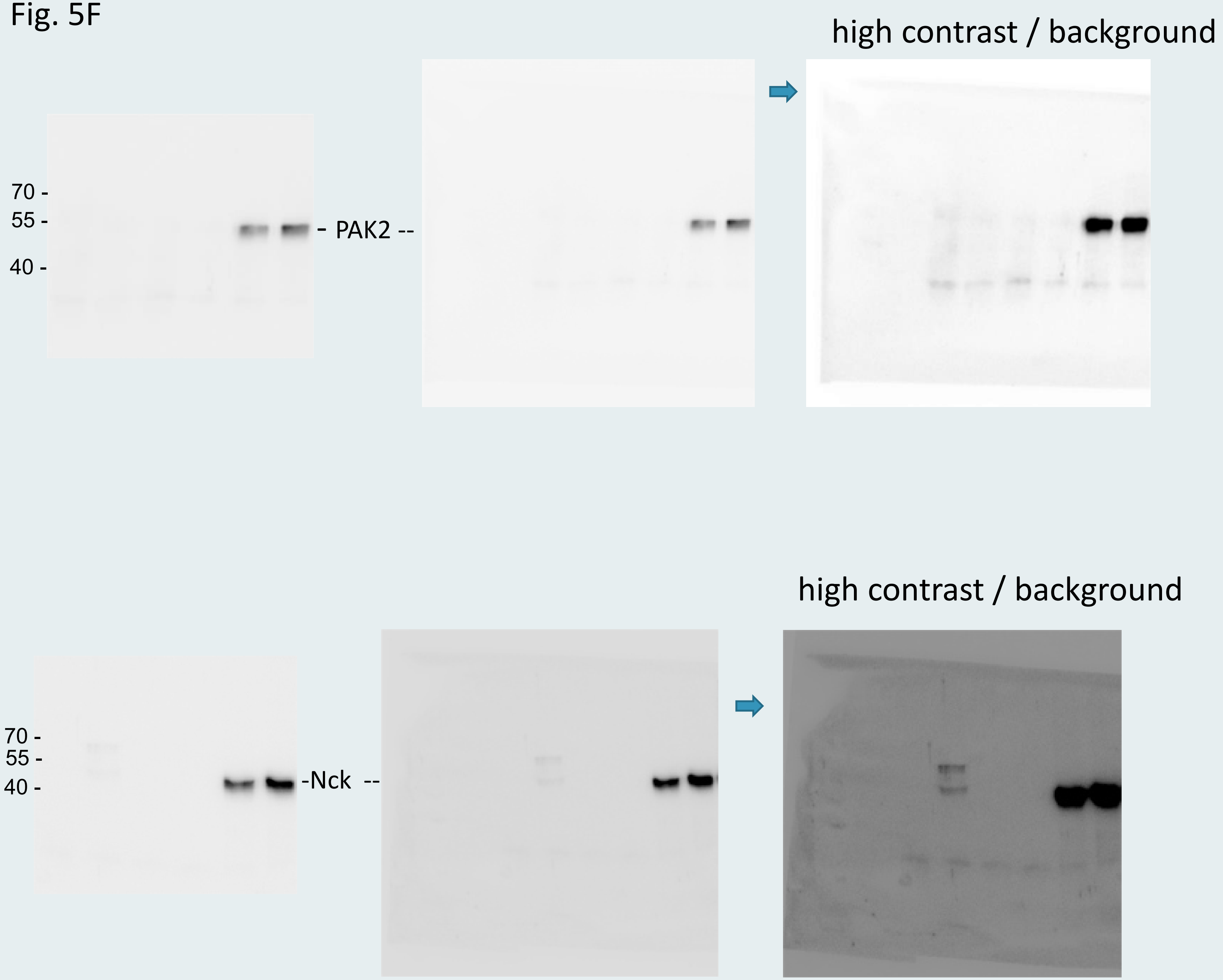

high contrast / background
70 -
55 -
40 -
- PAK2 --
high contrast / background
70 -
55 -
40 -
-Nck --

Fig. 5F

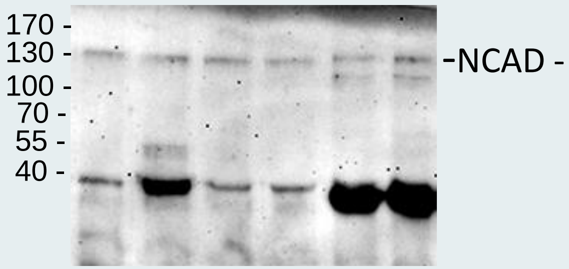

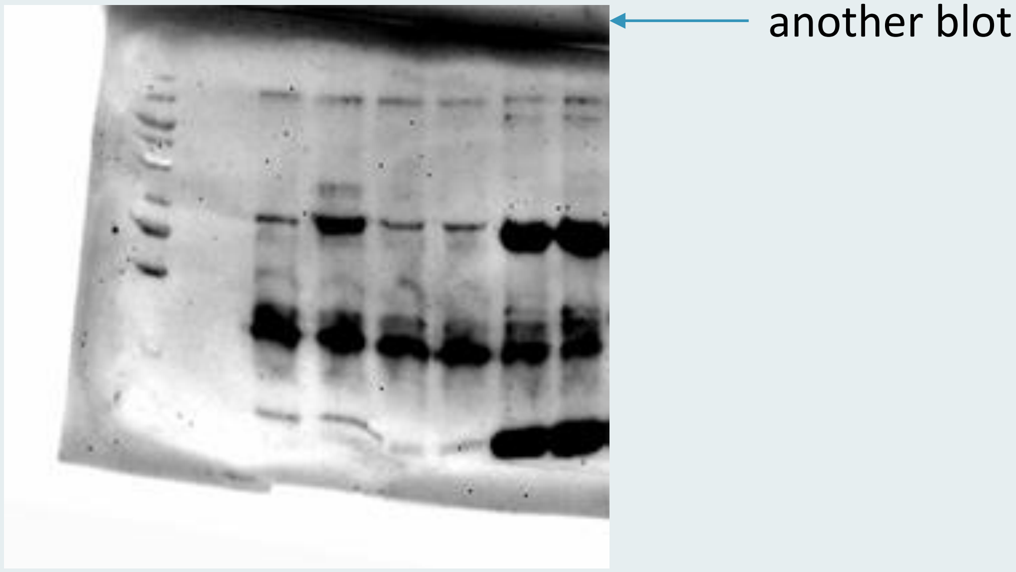

Fig. 6 B

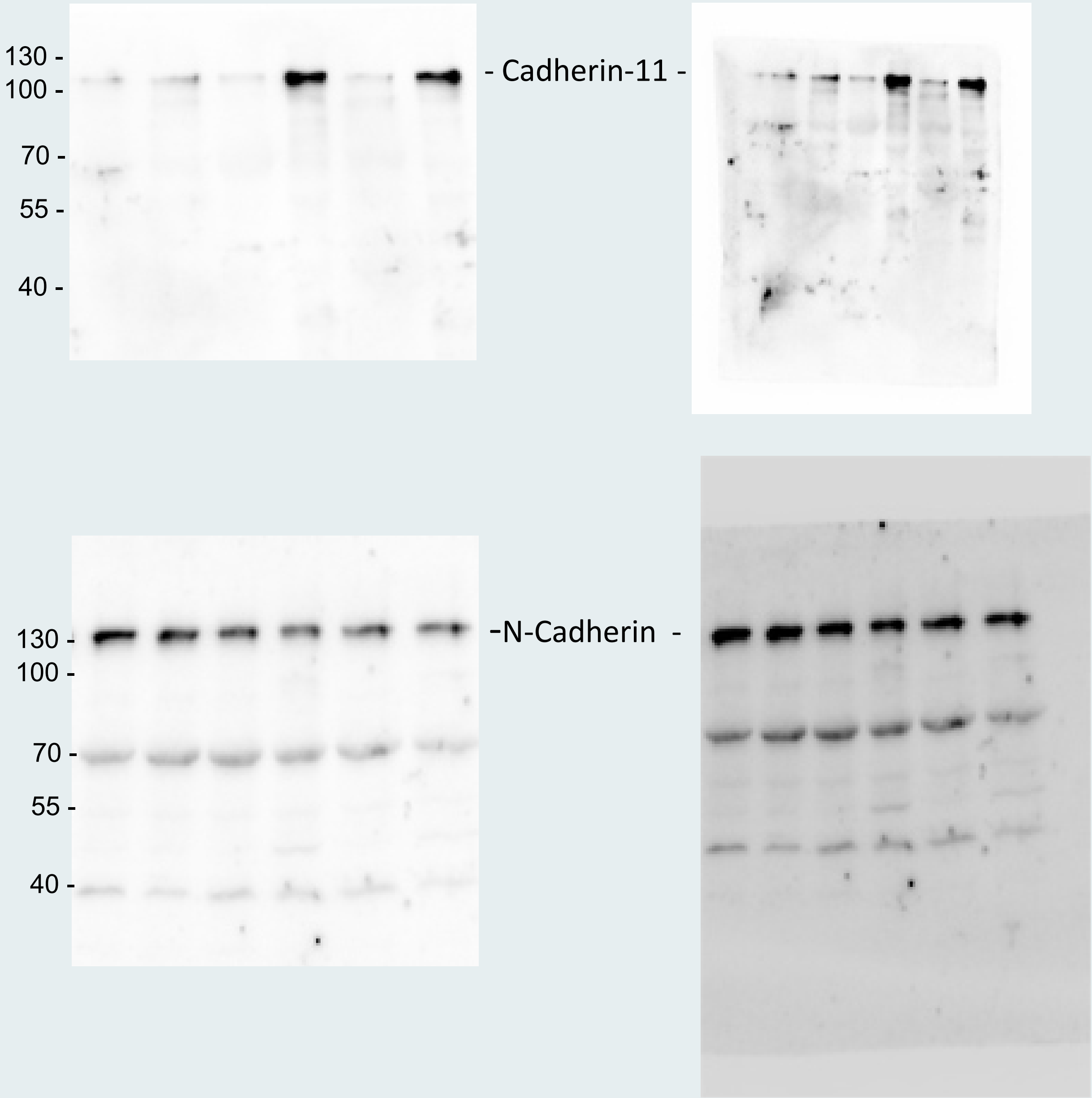

Fig. 6 B

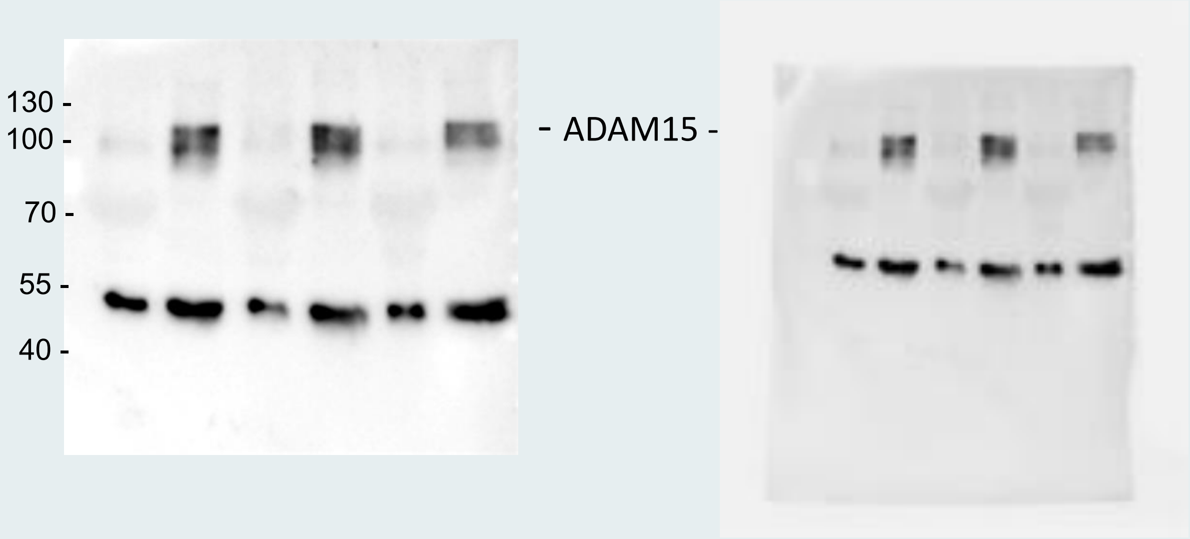

artificially merged image to show borders of blots

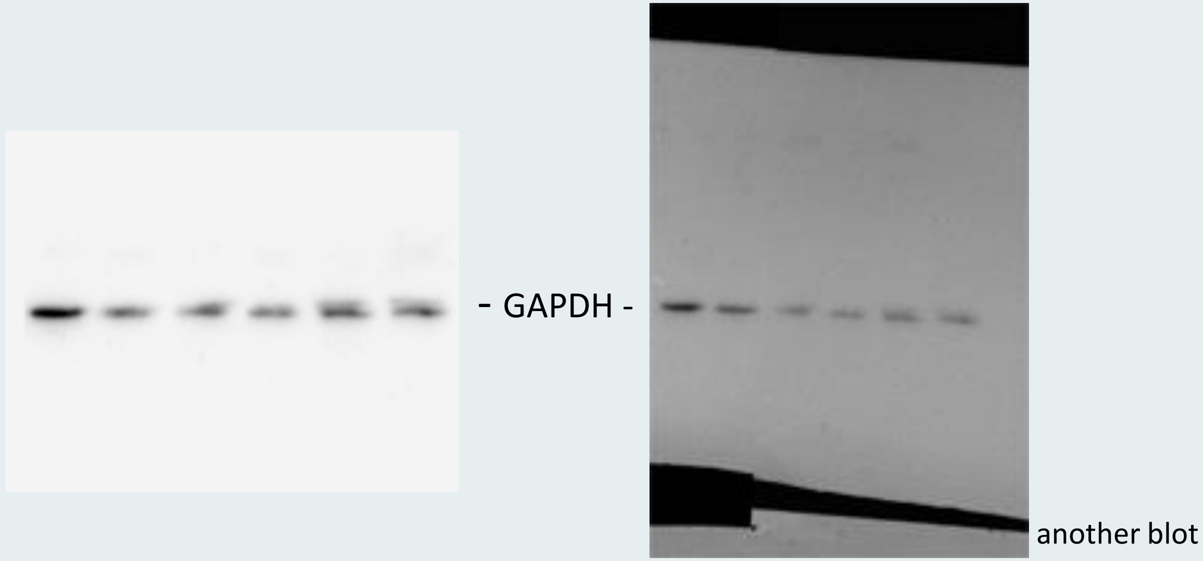

Fig. 6 D

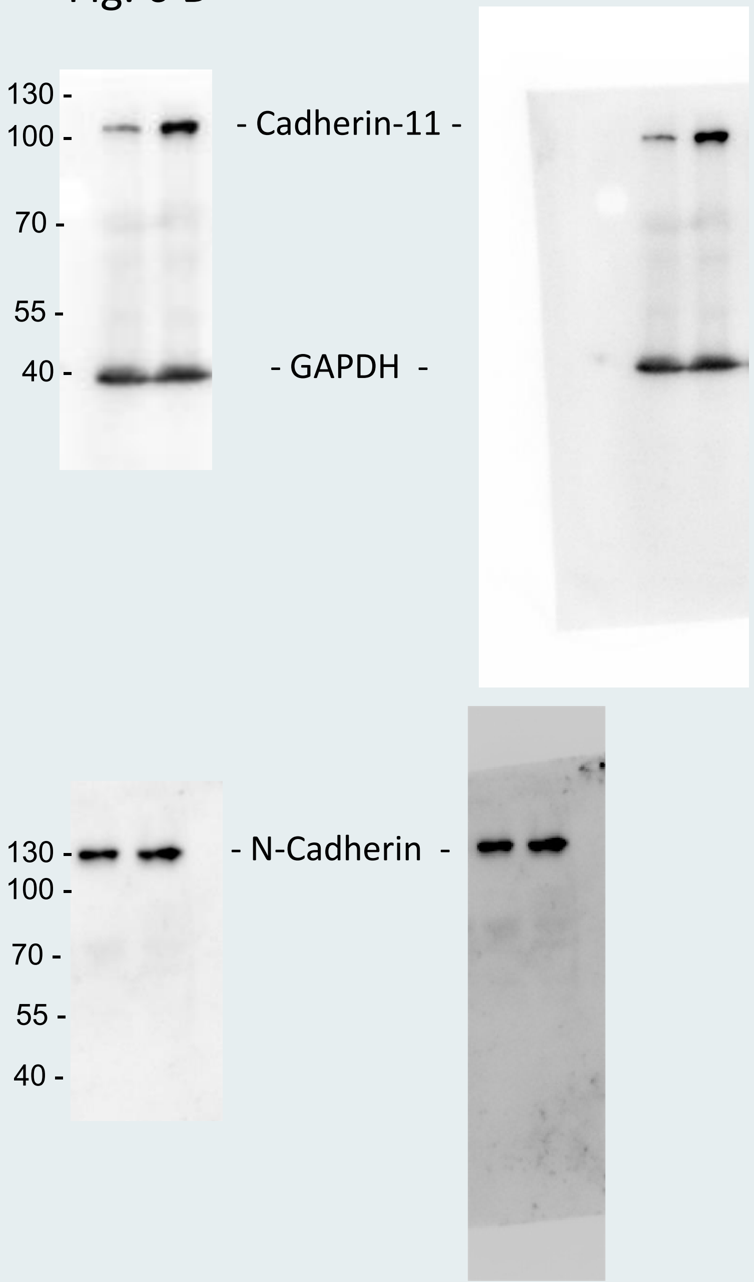

Fig. 7E

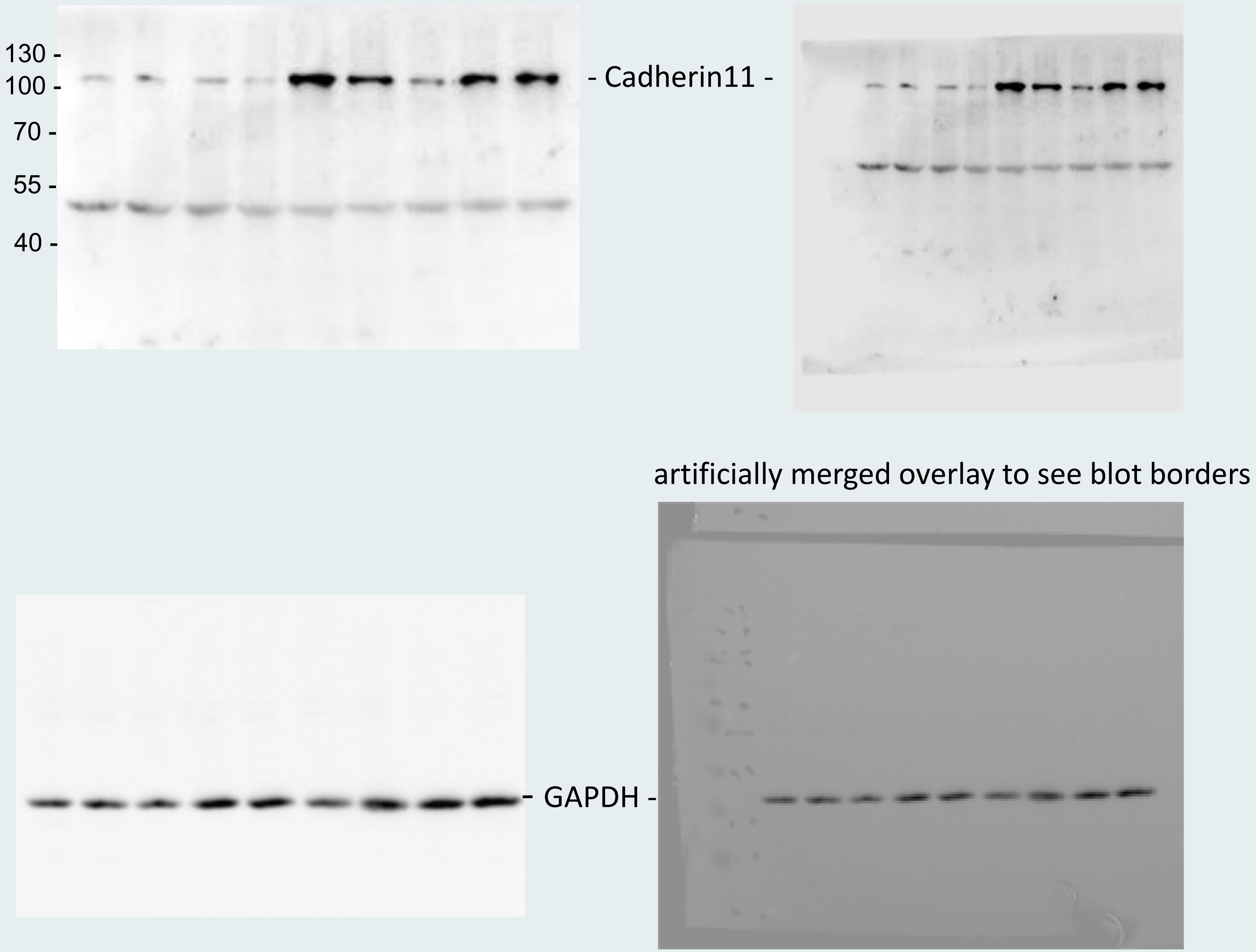
130 -
100 -
70 -
55 -
40 -
- Cadherin11 -
artificially merged overlay to see blot borders
- GAPDH -

Fig. 7E

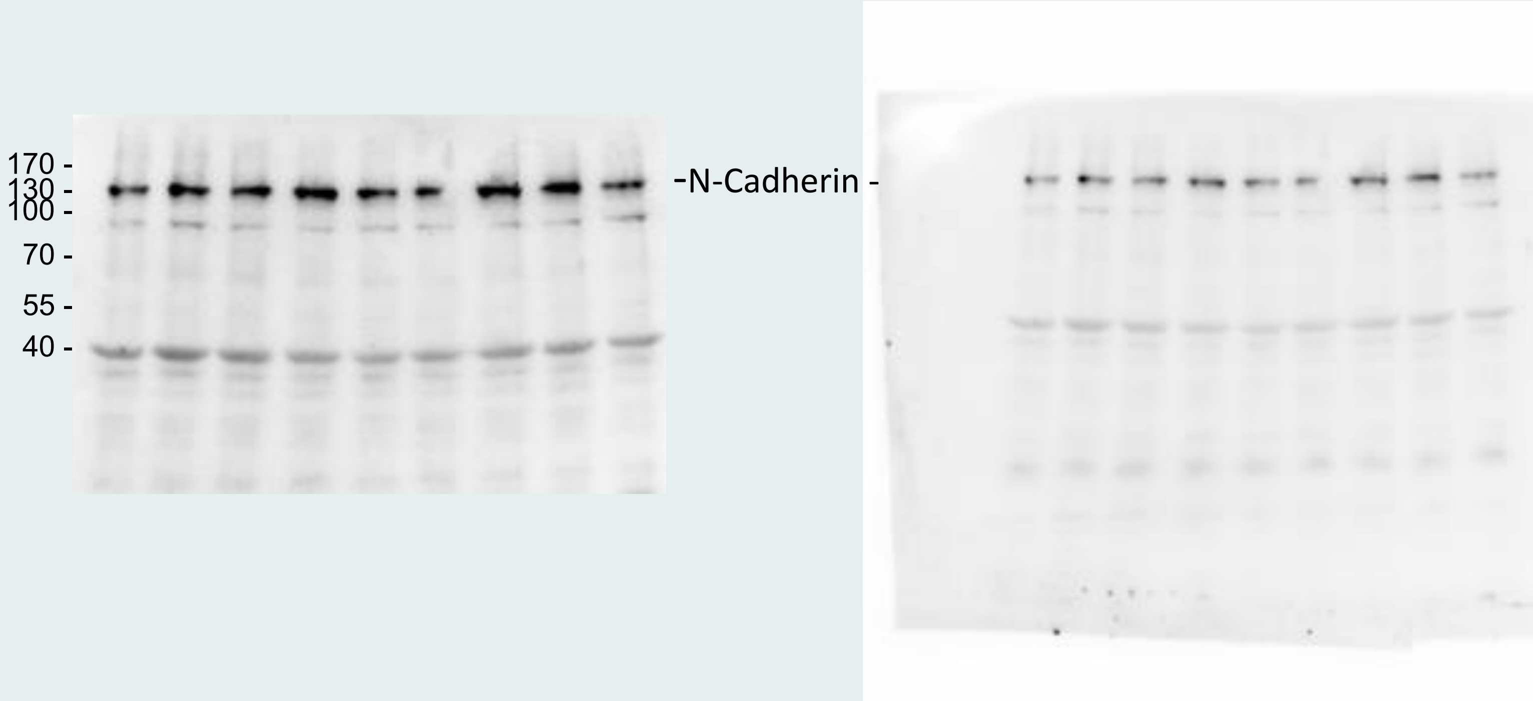

Fig. 8

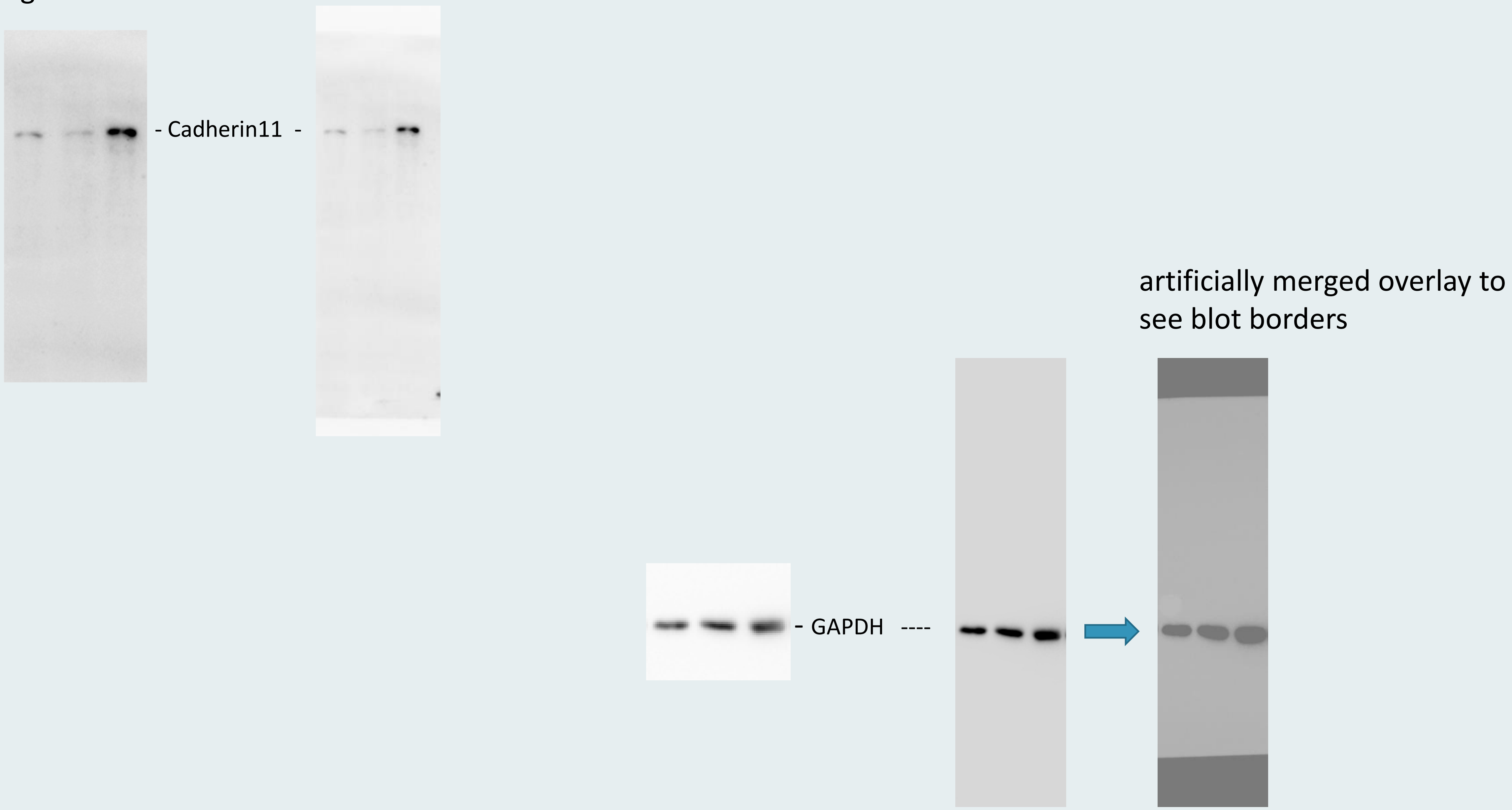
- Cadherin11 -
- GAPDH ----
artificially merged overlay to see blot borders

Supplementary figure file

Supplementary figure 2C

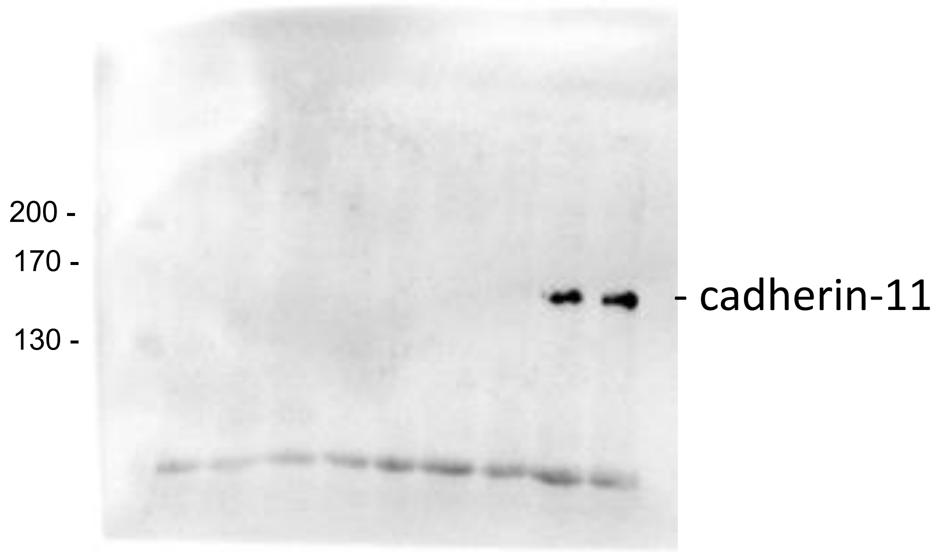

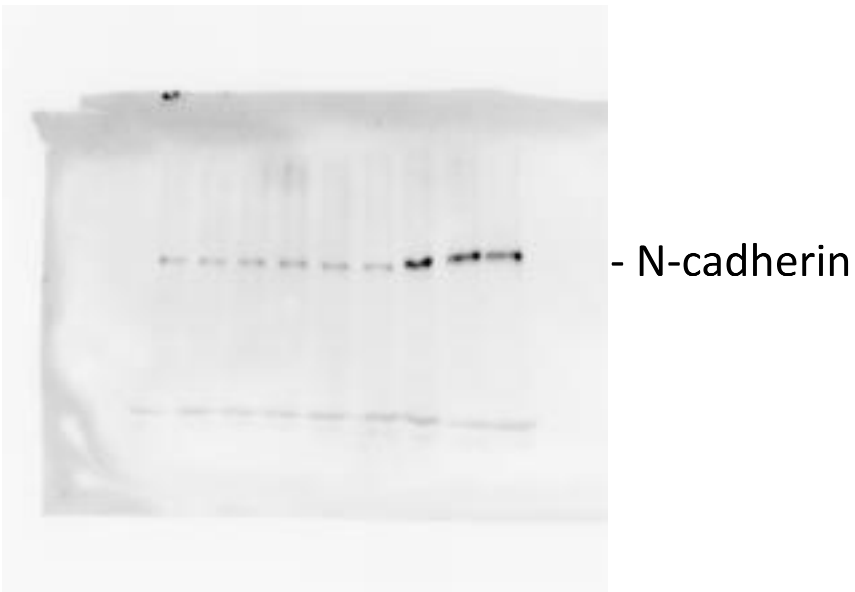

Supplementary figure file

Supplementary figure 2C

original

artificial merged overlay

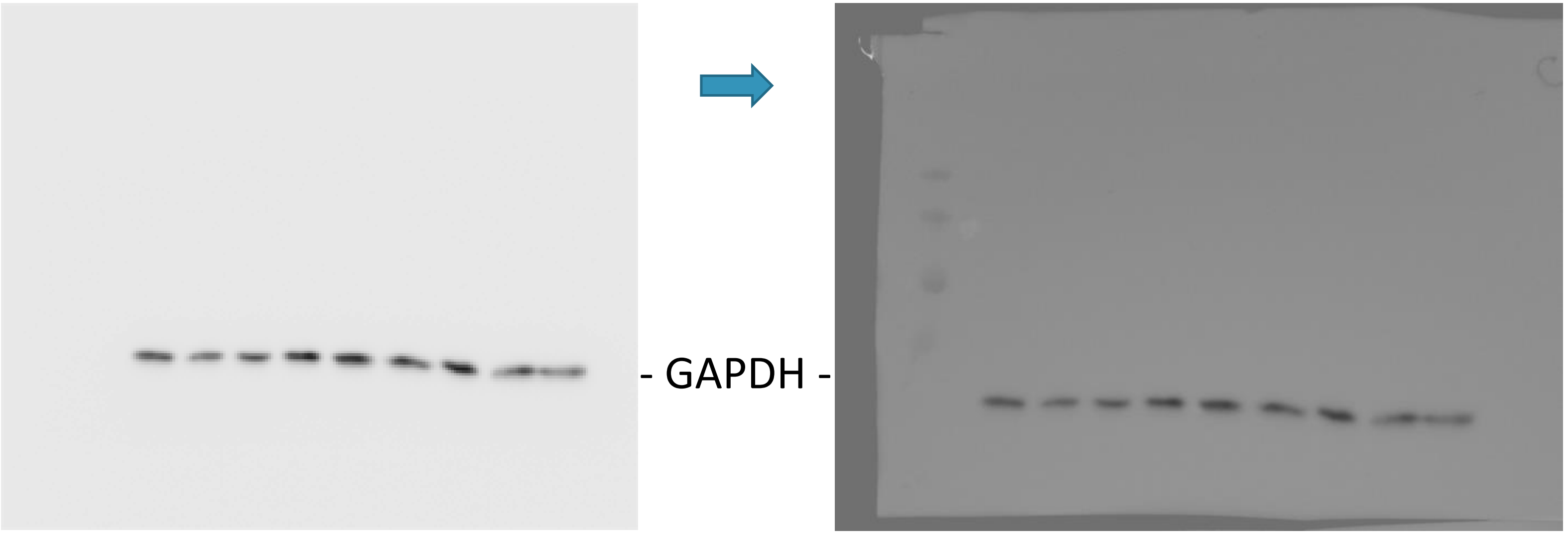

Supplementary figure file

Supplementary figure 3B

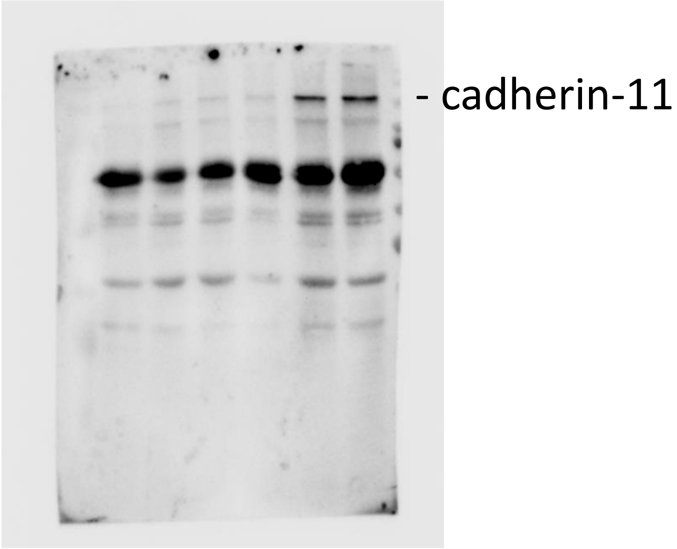

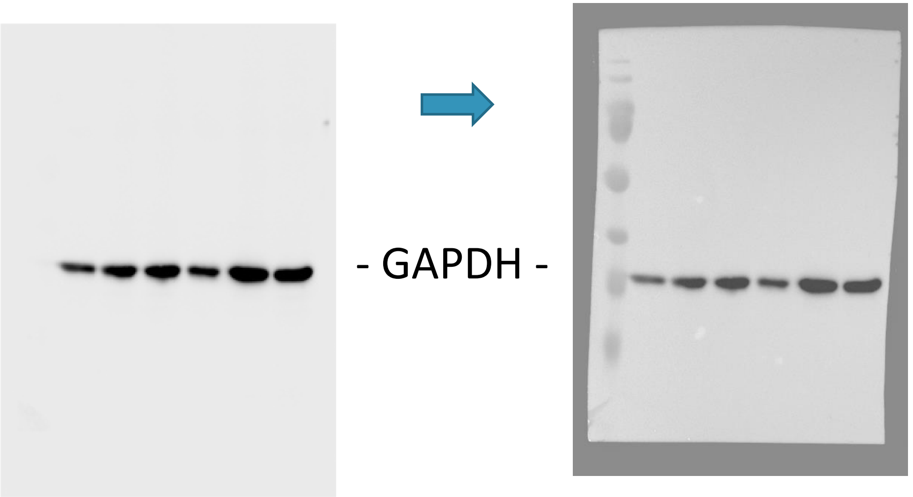

Supplement: Supplementary file 2 — Supplementary Material 2. [file 41598_2025_94012_MOESM2_ESM.pdf]
